# A tripartite therapeutic agent reprograms the myocardial infarction microenvironment

**DOI:** 10.1016/j.mtbio.2025.102600

**Published:** 2025-11-24

**Authors:** Kaiyi Zhu, Xiaozhe Wang, Qian Yang, Qiuyi Liao, Sunli Liu, Yuchen Cao, Wei Yang, Xueyan Li, Xiaolong Mi, Yuanyuan Lin, Qiang Zhou, Yue Song, Chunbo Dong, Yuping Gao, Zhida Liu, Liping Li, Ruiping Zhang

**Affiliations:** a Third Hospital of Shanxi Medical University, Shanxi Bethune Hospital, Shanxi Academy of Medical Sciences, Tongji Shanxi Hospital, Taiyuan, 030032, China; b Department of Neurosurgery, Beijing Children's Hospital, Capital Medical University, Beijing 100045, China; c Chinese Academy of Medical Sciences, Peking Union Medical College, Beijing 100050, China; d Shanxi Academy of Advanced Research and Innovation, Taiyuan 030032, China; e School of Basic Medical Sciences, Shanxi Medical University, Taiyuan 030001, China; f Radiology Department, Shanxi Provincial People's Hospital (Five Hospital of Shanxi Medical University), Taiyuan 030001, China

**Keywords:** Myocardial infarction, Conductivity, Antioxidant, Anti-inflammatory

## Abstract

Myocardial infarction (MI) remains a global health challenge despite advances in reperfusion therapies, as subsequent oxidative stress, inflammatory cascades, and electrical conduction abnormalities impede functional recovery. This study presents a tripartite therapeutic strategy addressing these pathological mechanisms through a clinically translatable multifunctional myocardial repair agent (MMRA). The system combines polypyrrole's and sinomenine's (Sino) anti-inflammatory/antioxidant effects, formulated within a conductive thermoresponsive Poloxamer 407 (P407) hydrogel platform. Injectable P407 hydrogel facilitates targeted delivery and sustained release of therapeutic components within the infarct zone. In vivo evaluations revealed MMRA's capacity to simultaneously: 1) Scavenge reactive oxygen species and downregulate pro-inflammatory cytokines, 2) Reprogram macrophage polarization toward tissue-reparative M2 phenotypes and 3) Restore electrical signal propagation through conductive hydrogel-mediated intercellular coupling. By synergistically modulating the post-infarction microenvironment while preserving myocardial electromechanical synchronization, this integrated therapeutic paradigm demonstrates significant potential for enhancing cardiac repair outcomes. The employment of clinically approved components (Sino and P407) further shows the excellent translational feasibility of the MMRA for MI treatment.

## Graphical abstract

**Figure:**
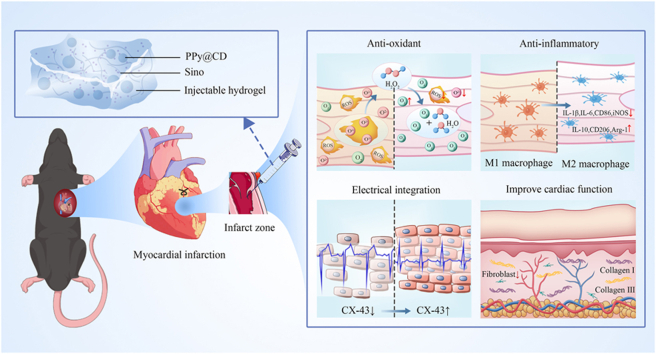

## 1 Introduction

Myocardial infarction (MI), caused by ischemia after blockage of the coronary arteries, is a leading cause of death [1]. MI results in severe myocardial injury and dysfunction, ultimately leading to heart failure [2]. Prompt and effective reperfusion of the infarcted myocardial tissue is essential for the preservation of cardiac function. Currently, traditional clinical treatments for MI primarily consist of percutaneous coronary intervention, coronary artery bypass grafting, and antiplatelet drug therapy [3]. While these interventions can partially restore blood supply to the affected tissue, they are insufficient reversing the damage and dysfunction after infarction, and may even exacerbate the myocardial microenvironment [[4], [5], [6], [7]]. Consequently, there is an urgent need for innovative therapeutic approaches to improve the prognosis of MI [8].

Among the various phases of MI pathogenesis, the myocardial microenvironment undergoes significant changes. Particularly, the inflammatory and oxidative stress phase holds particular significance due to its impact on subsequent healing and tissue remodeling [9]. During the inflammatory phase, the release of pro-inflammatory cytokines (e.g., IL-1β, IL-6) and damage-associated molecular patterns exacerbates inflammation and oxidative stress, initiating a cascade of immune responses that can further exacerbate tissue damage [10]. Excessive inflammation and reactive oxygen species (ROS) trigger the infiltration of neutrophils and monocytes, further resulting in scar formation, myocardial fibrosis, and maladaptive remodeling [11]. If the inflammatory and oxidative stress phases are effectively managed, this will contribute to slowing the progression of pathological remodeling, providing substantial protection for cardiac function, and reducing the risk of heart failure [12]. Although traditional oral or intravenous anti-inflammatory and antioxidant therapies can partially suppress inflammation, their effectiveness in optimizing the myocardial microenvironment remains limited. This is due to factors such as the continuous motion of the heart, poor drug retention in the affected area, and abnormal electrical signal conduction following MI [[13], [14], [15]]. Therefore, it is crucial to explore novel therapeutic strategies that can effectively regulate inflammation, oxidative stress, and conductivity after MI to improve cardiac function.

Hydrogel-based drug delivery systems have gained significant attention in the pathological process of MI due to their exceptional bioavailability, outstanding physiological stability, and electrical conductivity regulation capabilities [16]. Up to now, many hydrogel drugs based on inorganic and organic materials have been widely developed for the treatment of MI [17]. For example, Lee reported a conductive adhesive hydrogel heart patch made from two-dimensional titanium carbide (Ti_3_C_2_Tx) MXene combined with natural biocompatible polymers such as gelatin and dextran aldehyde, designed to aid in the recovery of infarcted heart function [18]. Wang and Zhu designed a hydrogel platform containing triptolide (TPL), which, upon myocardial injection, not only enhanced the anti-inflammatory and myocardial repair effects of TPL but also alleviated its hepatotoxicity and nephrotoxicity [19]. Although the aforementioned strategies effectively improve MI treatment, they primarily focus on either the regulation of myocardial tissue conductivity or the modulation of the myocardial microenvironment individually, neglecting the simultaneous integration of both. However, both conductivity regulation and microenvironment modulation are critical factors for cardiac repair.

**Scheme 1:**
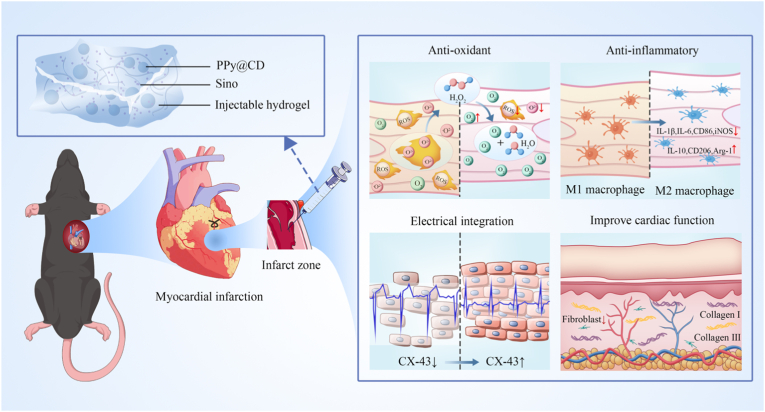
Schematic illustration of MMRA treatment for MI via anti-inflammatory, antioxidative, and conductive properties for myocardial repair.

Herein, we developed an injectable, highly biocompatible multifunctional myocardial repair agent (MMRA) that integrates anti-inflammatory and antioxidant properties, conductivity, and thermosensitivity, designed to synergistically regulate the myocardial microenvironment and electrical conduction for MI treatment. MMRA consists of polypyrrole (PPy, good biocompatibility), sinomenine (Sino, FDA-approved anti-inflammatory alkaloid), and thermoresponsive Poloxamer 407 hydrogel (P407, clinically used drug carrier). PPy can scavenge free radicals, enhance cell adhesion and migration, and improve cell-material interactions [[20], [21], [22], [23], [24]]. Sino is a monomer derived from traditional Chinese medicine, has been approved for the treatment of rheumatoid arthritis, with multiple biological functions, including anti-inflammatory, antioxidant, as well as the ability to promote macrophage polarization toward the tissue-reparative M2 phenotype [[25], [26], [27]]. Importantly, it is potential to treat MI [27]. In addition, the use of the thermosensitive injectable hydrogel P407 provides a longer myocardial retention time compared to free Sino solution, thereby extending the *in vivo* half-life of Sino, and reduces the risk of allergic reactions, intestinal side effects, and blood toxicity [28,29]. At the same time, P407 also provides the necessary electrical conductivity for myocardial repair, promotes synchronized myocardial contraction [30]. In summary, MMRA effectively improves the MI treatment microenvironment through a triple strategy: scavenging reactive oxygen species and downregulating pro-inflammatory cytokines, reprogramming macrophage polarization toward the tissue-reparative M2 phenotype, and restoring electrical conductivity between damaged cardiomyocytes (see Scheme 1). This work provides a multifunctional MMRA with excellent injectability, conductivity, anti-inflammatory, and antioxidant properties, offering a collaborative approach with clinical translation potential for the synergistic treatment of MI.

## 2 Results and discussion

### 2.1 Synthesis and characterization of MMRA

**Fig. 1:**
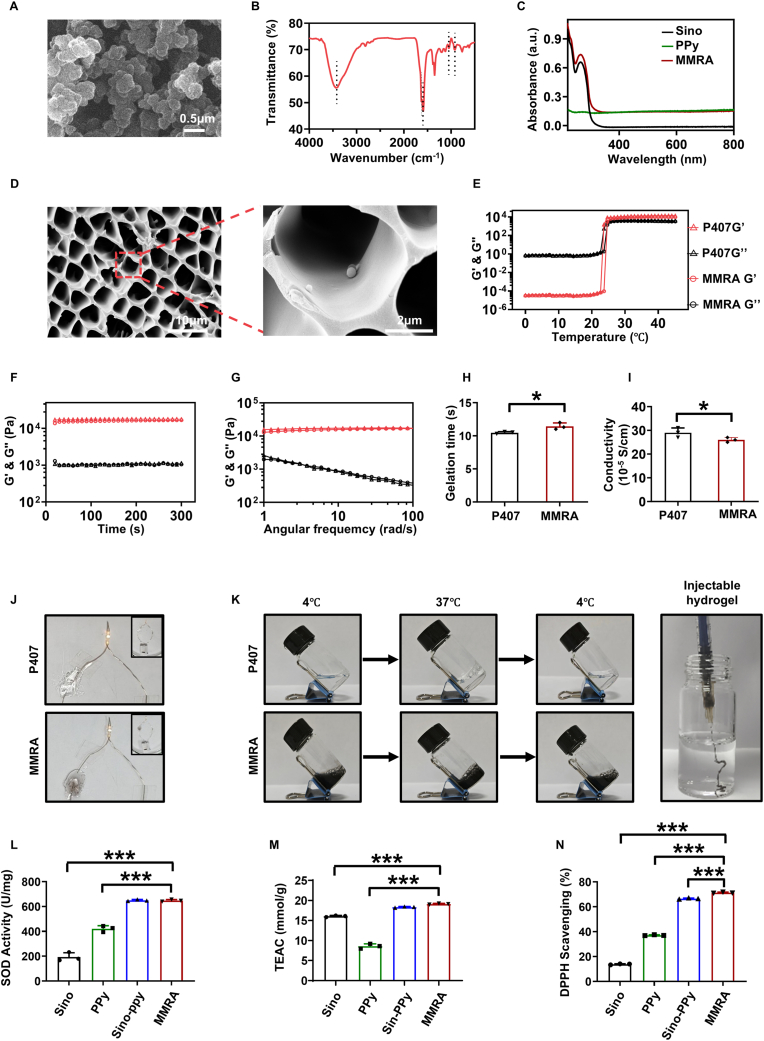
Synthesis and characterization of MMRA. (A) SEM image of PPy. (B) FTIR spectra of PPy. (C) UV-Vis-NIR spectra of Sino, PPy and MMRA. (D) SEM images of MMRA under different magnifications. (E) Rheological behavior of P407 hydrogel and MMRA under elevated temperature conditions. (F–G) Rheological analysis of the hydrogels at 37 °C in time sweep mode and amplitude sweep mode. (H) Gelation time of P407 and MMRA (n = 3). (I) Conductivity of P407 and MMRA (n = 3). (J) Photographs of an LED circuit with P407 and MMRA. (K)The sol−gel−sol transition of P407 and MMRA at various temperatures; Photograph of the MMRA injected into DI water through a needle. (L–N) SOD activity, ABTS and DPPH scavenging activities of Sino, PPy, Sino-PPy solution and MMRA. ∗*P* < 0.05, ∗∗*P* < 0.01, ∗∗∗*P* < 0.001.

Firstly, PPy was synthesized by chemical oxidation using pyrrole as monomer, ammonium persulfate as oxidant and cyclodextrin as stabilizer. The combined analysis by scanning electron microscopy (SEM) and transmission electron microscopy (TEM) demonstrated that the PPy exhibited discrete, amorphous, and homogeneous distribution, along with uniformly spherical morphology and well-defined contours (Fig. 1A, Fig. S1A). The Fourier transform infrared (FTIR) spectra showed the characteristic peaks of PPy, that is 3410 cm^−1^ (NH stretching vibration), 1630 cm^−1^ (stretching vibration in pyrrole ring with C=C bonding), 1048 cm^−1^ (in-plane stretching vibration of C-H and N-H bonds) and 928 cm^−1^ (stretching vibration of C-C bond) [[31], [32], [33]] (Fig. 1B). The UV-Vis-NIR spectra of PPy showed broad absorption bands in the wavelength range of 200–800 nm (Fig. 1C). MMRA was formed by mixing Sino and PPy in 20 % w/v P407 solution at 4 °C. As shown in Fig. 1C, the MMRA solution has the characteristic absorption peak of the Sino solution at 264 nm as well as the broad spectrum absorption of the PPy solution. SEM examination of the P407 hydrogel showed that the P407 hydrogel had a macroporous structure, which was favorable for the encapsulation of the drug (Fig. S1B). The MMRA and P407 hydrogel had similar gel networks, and the spherical PPy can observed in the gel network of MMRA (Fig. 1D). The rheological properties of the hydrogels were tested using a rotational rheometer. The effect of temperature on the mechanical properties of P407 hydrogel and MMRA were first evaluated. As shown in Fig. 1E, the addition of Sino and PPy did not significantly change the modulus of P407 hydrogel, and the critical temperatures for the phase transitions of both P407 hydrogel and MMRA were 23.5 °C. The evaluation of gel kinetics in dynamic time scanning mode is shown in Fig. 1F, and the storage modulus (G′) values of both P407 and MMRA were consistently larger than the loss modulus (G″) values within 5 min. The gel dynamics evaluated in the amplitude scanning mode are shown in Fig. 1G, with the increase of angular frequency from 1 rad/s to 100 rad/s, the G′ of both P407 and MMRA was larger than G″, indicating the formation of a stable colloidal system. The gelation time of hydrogels was examined by the “tilt method”. The P407 solution gelled rapidly at about 10 s under the action of a 37°C-water bath, and the gelation time of the MMRA solution was slightly increased by the addition of Sino and PPy (Fig. 1H). As shown in Fig. 1K, the MMRA solution gelled rapidly after injection into 37 °C water. The electrical conductivity of the hydrogel is critical for the transmission of electrical signals in the infarct zone [34]. The conductivity of the hydrogel was measured using an AC four-probe resistivity tester (Fig. 1I). We were surprised to find that the conductivity of the P407 hydrogel at 20 % w/v alone was 2.9 × 10^−4^ S/cm, whereas the addition of Sino and PPy resulted in a conductivity of 2.6 × 10^−4^ S/cm for MMRA, which is in line with the conductivity range of the natural cardiac tissues (approximately 10^−4^ S/cm) [18]. In addition, we chose simple circuit experiments to verify the electrical signal transfer ability of P407 hydrogel and MMRA. The results showed that both P407 hydrogel and MMRA could conduct electrical signals inducing obvious light bulb luminescence, indicating the good electrical conductivity of P407 hydrogel and MMRA (Fig. 1J). These results indicate that MMRA with gel properties effectively mimics the conductive microenvironment of native cardiac tissue, laying the foundation for further enhancing electrical conductivity and integration in the infarcted region and ultimately promoting cardiac regeneration.

The microenvironment after MI is characterized by an overproduced and uncontrolled inflammatory condition [9]. Superoxide dismutase (SOD) has a high content in cardiac tissues and its main role is to scavenge free radicals *in vivo* [35]. The function of free radical scavenging system decreases when cardiomyocytes are ischemic and hypoxic, and the free radicals cause acute or chronic damage to the tissues in various ways [36]. Hydrogels with SOD enzyme activity are essential for post-infarction repair. After confirming the gel properties and electrical conductivity of MMRA, we evaluated the antioxidant properties of MMRA in vitro. As shown in Fig. 1L, both Sino and PPy possessed SOD enzyme activities, and both activities were not significantly affected in the gel. To further verify the ROS scavenging ability of MMRA, we examined its scavenging activity against 2,2′-azido (3-ethylbenzothiazoline-6-sulfonic acid) (ABTS) radical and 1′-diphenyl-2-pyridinamide radical (DPPH). The results showed that MMRA had high scavenging ability for both ABTS and DPPH (Fig. 1M and N). MMRA's antioxidant property is beneficial for removing various types of harmful ROS in the infarct region and remodeling the infarct microenvironment.

### 2.2 MMRA protects myocardial cells in an in vitro inflammatory and oxidative microenvironment

First, we tested the cell viability and LDH release of H9C2 cells treated with different concentrations of MMRA and normal or H_2_O_2_ conditions. As shown in Fig. S2A-D, 50 μg/mL MMRA had no significant effect on the viability and LDH release of normal H9C2 cells, and exhibited significant antioxidant ability under H_2_O_2_ conditions. Therefore, in subsequent experiments, the default concentration of MMRA is 50 μg/mL.

**Fig. 2:**
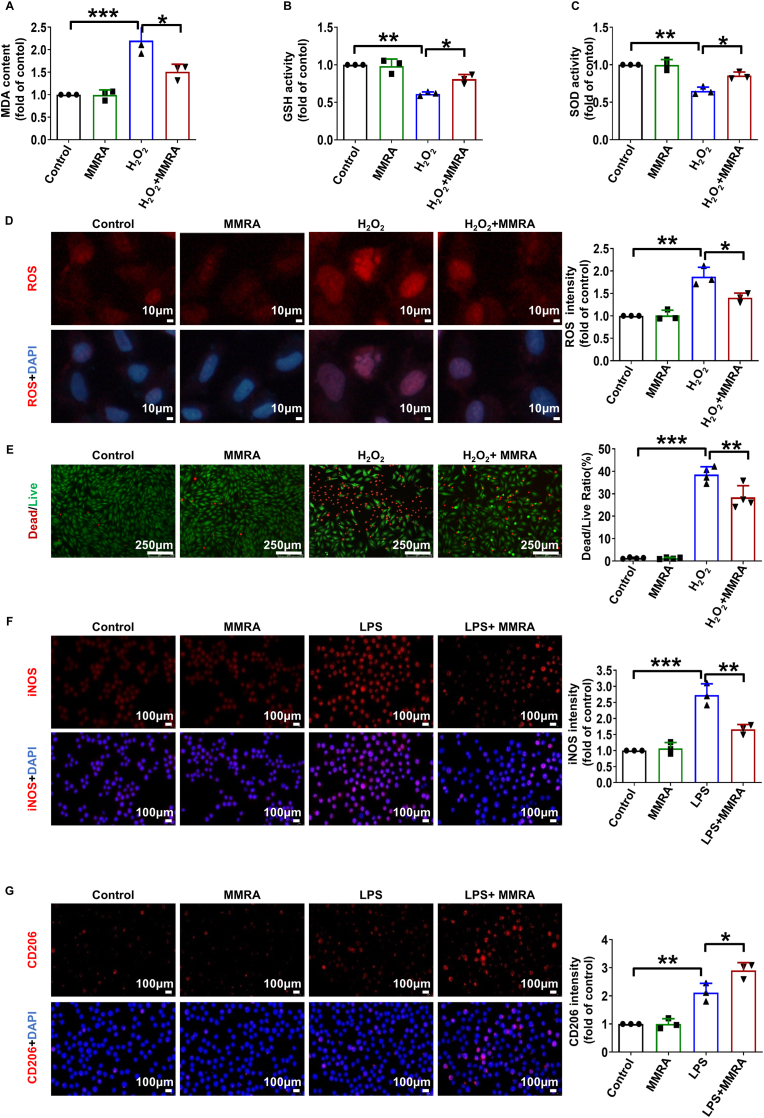
The protective effects of MMRA on H_2_O_2_-induced oxidative stress and LPS-induced inflammation. (A–C) The level of MDA, GSH and SOD in H9C2 cells exposed to H_2_O_2_ (n = 3). (D) The ROS levels were determined by DHE (red) staining in H9C2 cells (n = 3). (E) Live/dead staining in H9C2 cells (n = 4). (F, G) Representative immunofluorescence staining of iNOS and CD206 in the LPS-induced inflammation in RAW264.7 cells (n = 3). ∗*P* < 0.05, ∗∗*P* < 0.01, ∗∗∗*P* < 0.001.

MI-induced excess ROS leads to oxidative stress injury at the site of injury, resulting in cardiomyocyte death, which can be simulated in vitro by placing H9C2 cells in a culture medium containing H_2_O_2_ to create an artificial oxidative stress environment [37]. Notably, Sino has been reported to exert protective effects by modulating cell death, inflammation, calcium overload, and oxidative stress [38,39]. Malondialdehyde (MDA), superoxide dismutase (SOD), and glutathione (GSH) levels are widely used as biomarkers to assess oxidative stress. MDA levels were increased in the H_2_O_2_ group, while SOD and GSH levels were decreased (Fig. 2A–C). However, MDA levels were significantly decreased, and SOD and GSH levels were significantly increased in the H_2_O_2_+MMRA group (Fig. 2A–C). The antioxidant properties and effects of MMRA in oxidative stress environment were further validated. The ROS fluorescence intensity was significantly increased in H_2_O_2_ group, which was markedly reduced in the H_2_O_2_+MMRA group (Fig. 2D). Live/dead staining further revealed that MMRA was non-cytotoxic to H9C2 cells under normal conditions and significantly alleviated H_2_O_2_-induced cell damage (Fig. 2E).

Connexin 43 (CX43), a gap junction protein in cardiomyocytes, is closely related to the conduction of electrical signals between cells and the propagation of action potentials [40,41]. The α-actinin is a cardioskeletal protein closely associated with the synchronous contraction of cardiomyocytes [42]. In the study by Song et al., PPy significantly promoted cardiomyocyte growth and facilitated the formation of highly oriented sarcomere structures, thereby accelerating the maturation of synchronized contraction function [43]. Similarly, Immunofluorescence staining of CX43 and α-actinin was used to assess the electrical integration of H9C2 cells treated with MMRA, and the results indicated that MMRA effectively restored the expression of CX43 and α-actinin in cells with H_2_O_2_-induced damage (Fig. S3A and B). These results indicate that MMRA has obvious antioxidant properties in H_2_O_2_-induced oxidative stress injury, promoting the recovery of the contractile function of cardiomyocytes and the gap junction intercellular communication, and finally mitigating H_2_O_2_-induced cell damage.

The phenotypic transformation of macrophages, transitioning from the pro-inflammatory M1 phenotype to the anti-inflammatory or pro-healing M2 phenotype, plays a pivotal role in mitigating chronic inflammation and facilitating tissue repair [16]. The polarization state of macrophages is not strictly limited to the M1 or M2 phenotype but instead exhibits characteristics of both, dynamically regulated by the surrounding microenvironment [44]. Recent study has confirmed that the characteristics of biomaterials can significantly influence macrophage phenotypes. In the heparin-co-doped PPy electrode system, macrophages exhibit a pronounced tendency toward anti-inflammatory phenotype polarization [45]. This phenomenon suggests that the PPy component is a favorable factor in promoting macrophage polarization toward an anti-inflammatory phenotype. In addition, Sino acts on the α7 nicotinic acetylcholine receptor (α7nAChR) to inhibit the inflammatory response. This is achieved by downregulating α7nAChR through the α7nAChR/ERK/Egr-1 feedback pathway, thereby suppressing macrophage M1 polarization and the inflammatory response [46]. To investigate the effect of MMRA on macrophage polarization, an LPS-induced inflammatory model using RAW264.7 cells was established. Immunofluorescence staining, flow cytometry, and ELISA were employed to evaluate M1/M2 phenotypic changes and cytokine secretion. As shown in Fig. 2F and G, MMRA not only suppressed the expression of iNOS, a molecular marker of M1 macrophages, but also enhanced the expression of CD206, a characteristic marker of M2 macrophages. Under LPS stimulation, CD86 and CD206 were used as markers to identify polarized macrophages by flow cytometry. As illustrated in Fig. S4, LPS stimulation markedly increased the proportion of CD86^+^ macrophages to 51.9 % within the total cell population, whereas MMRA treatment reduced this proportion to 32.2 %. Notably, in the LPS + MMRA group, the proportion of CD206^+^ M2 macrophages increased to 22.8 %, which was markedly higher than that in both the control and LPS groups, indicating that MMRA promoted macrophage polarization toward an anti-inflammatory M2 phenotype. ELISA analysis further demonstrated that MMRA modulated cytokine secretion in LPS-stimulated RAW264.7 cells. Specifically, the levels of IL-1βand TNF-α were decreased in the LPS + MMRA group, whereas the levels of IL-10 and TGF-β was significantly increased compared with the LPS group (Fig. S5A–D). These findings suggest that MMRA has the potential to promote changes in the inflammatory phenotype of macrophages in vitro.

### 2.3 MMRA treatment improves cardiac function *in vivo*

**Fig. 3:**
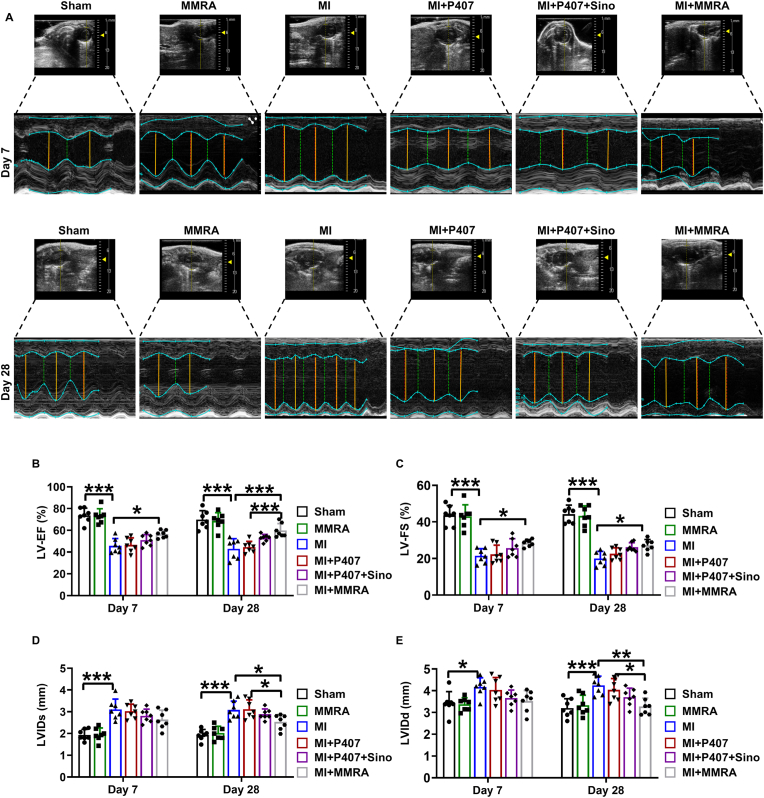
MMRA treatment improves cardiac function in MI mice. (A) Representative images of cardiac echocardiography. (B–E) Quantitative analysis of LVEF%, LV-FS%, LVIDs and LVIDd (n = 7). ∗*P* < 0.05, ∗∗*P* < 0.01, ∗∗∗*P* < 0.001.

MI typically leads to a significant decline in the heart's pumping ability, resulting in the deterioration of left ventricular function, which can ultimately progress to heart failure [47]. Early restoration of cardiac function is crucial for reducing the risk of long-term adverse cardiovascular events and improving overall survival rates. Therefore, therapeutic strategies aimed at alleviating cardiac dysfunction are importance for improving patient prognosis. To further clarify the role of MMRA, a more detailed evaluation was performed in a mouse MI model. First, echocardiogram analysis was used to assess the contractile cardiac function in each group. In mice at days 7 and 28 post MI, left ventricular ejection fraction (LV-EF%) and left ventricular fractional shortening (LV-FS%) were significantly decreased in MI group, indicating the deterioration in cardiac function and the occurrence of MI (Fig. 3A–C). However, LV-EF% and LV-FS%, two important indicators of heart pumping function, were most significantly improved in MI + MMRA group compared to other treatment groups (Fig. 3A–C). Significantly increased left ventricular internal diameter in diastole (LVIDd) and left ventricular internal diameter in systole (LVIDs) in MI group were also most effectively reversed in MI + MMRA group, suggesting that MMRA effectively inhibited MI (Fig. 3D and E). The results of the cardiac function assessment clearly indicate that MMRA can help improve cardiac dysfunction after MI.

### 2.4 MMRA treatment reduces cardiomyocyte apoptosis *in vivo*

**Fig. 4:**
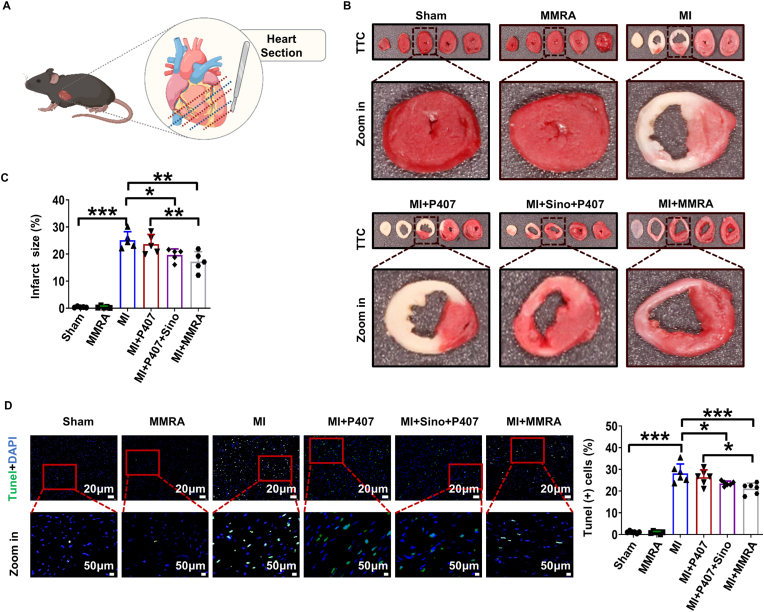
MMRA treatment reduces infarct size. (A)TTC staining schematic diagram of tissue sections. (B–C) Representative tissue sections and quantitative analysis of infarct area (n = 5). (D) TUNEL staining of myocardial tissue (n = 6). ∗∗*P* < 0.01, ∗∗∗*P* < 0.001.

Sino can reduce cell apoptosis through its anti-inflammatory and antioxidant effects [48]. PPy can effectively promote the regeneration, repair, and activation of cellular functions in electrically sensitive tissues such as bone, nerve, and myocardial tissues under electrical stimulation [49]. MMRA innovatively integrates the electrophysiological regulatory properties of P407 with the multidimensional protective mechanisms of Sino and PPy, which counteract oxidative stress and inhibit inflammatory pathways. By restoring myocardial electrical signal transmission and synergizing with the radical scavenging effects of bioactive molecules, MMRA forms a cascade protection strategy that inhibits cardiomyocyte apoptosis. TTC staining of cardiac tissues indicated that the MI + MMRA group exhibited the most significant reduction in myocardial infarct area among all treatment groups, including MI + P407 and MI + Sino + P407, when compared to the MI group (Fig. 4A–C). Terminal deoxynucleotidyl transferase dUTP nick-end labeling (TUNEL) is a widely used method for detecting and quantifying apoptotic cells in MI tissue. TUNEL analysis revealed a significant increase in cardiomyocyte apoptosis in the MI group. Compared with the MI group, the MI + Sino + P407 group exhibited a marked reduction in apoptosis, whereas the MI + MMRA group showed an even more pronounced anti-apoptotic effect (Fig. 4D). These results indicate that treatment of MI mice with MMRA significantly reduced cell apoptosis, and promoted myocardial repair.

### 2.5 Antioxidant and electrical integration of MMRA in the MI region

**Fig. 5:**
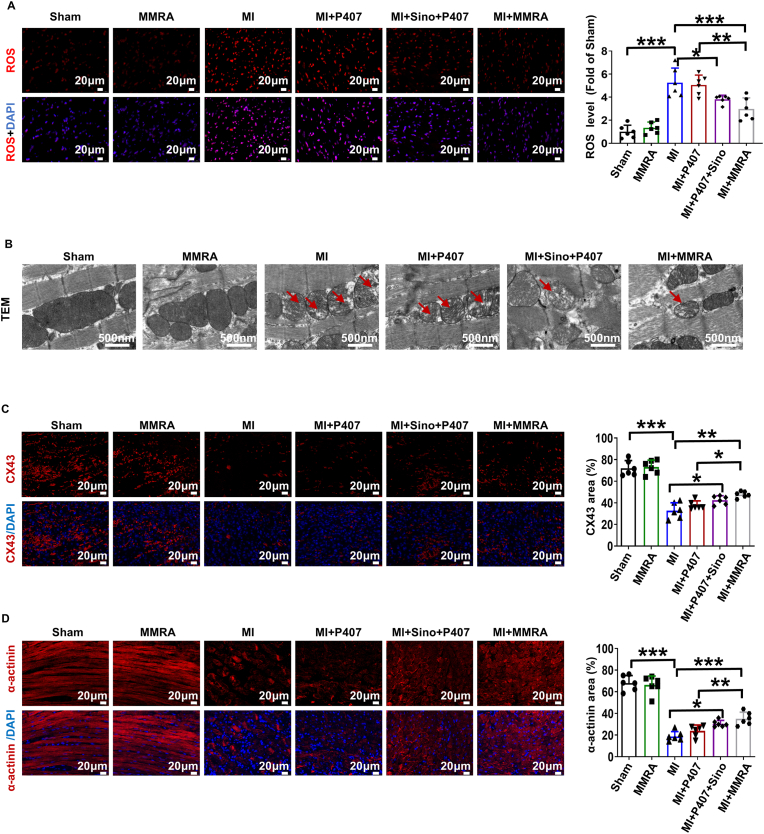
Antioxidant and electrical integration effects of MMRA after MI. (A) Representative DHE staining images and quantitative analysis of ROS level (n = 6). (B) Representative images of mitochondrial morphology using TEM. (C–D) Representative fluorescence images and quantitative analysis of CX43 and α-actinin (n = 6). ∗*P* < 0.05, ∗∗*P* < 0.01, ∗∗∗*P* < 0.001.

ROS are a major cause of mitochondrial damage, and their excessive accumulation in the myocardium following MI exacerbates this damage [16,50]. Mitochondria not only provides the primary energy supply for cardiomyocytes but also plays a pivotal role in regulating apoptosis [51]. During MI, the overproduction of ROS not only accelerates cardiomyocyte death but also impairs electrical signal transmission and disrupts the synchronous contraction of myocardial tissue [16,50,52]. Therefore, we further evaluated the effect of MMRA treatment at the injury site on ROS levels and mitochondria after MI. As shown in Fig. 5A, significant fluorescence signals were detected in the MI group and attenuated in the MI + Sino + P407 and MI + MMRA group, indicating that MMRA could exert antioxidant property and reduce accumulation of ROS in the infarct region. Observations of the mitochondrial morphology in cardiomyocytes from all groups using TEM revealed that the MI group exhibited loss of mitochondrial cristae and vacuolization compared to the Sham group, while Sino + P407 and MMRA treatment mitigated these structural damages (Fig. 5B). These results indicate that MMRA has antioxidant properties, and treatment with MMRA loading can significantly reduce oxidative stress and improve mitochondrial damage after MI.

At the cellular level, the expression of CX43 and α-actinin has been validated to be closely associated with cardioprotection and synchronized contraction of cardiomyocytes. Therefore, we examined their expression in the MI model. Staining demonstrated that, compared with the MI group, all three treatment groups showed an increased abundance of CX43 in the injured myocardium, with the MI + MMRA group exhibiting a more pronounced elevation (Fig. 5C). It also showed that the area coverage of α-actinin was significantly lower in the MI group, whereas MMRA treatment in MI + MMRA group recovered its expression in injured myocardial tissues (Fig. 5D). Electrocardiogram (ECG) analysis further confirmed the electrophysiological repair capacity of MMRA (Fig. S6). At 3–7 days post-operation, pathological Q waves, QRS complex widening, and abnormal T waves were observed in the MI group, indicating impaired electrical coupling (Fig. S6). These pathological changes were partially alleviated in the MI + P407 and MI + Sino + P407 groups. Notably, the MI + MMRA group exhibited a significant improvement in QRS duration and effectively promoted ST segment recovery. These results collectively suggest that MMRA could restore myocardial electrical conduction function and improve the systolic and diastolic functions of the heart in MI by enhancing electrical coupling within the infarcted tissue.

### 2.6 MMRA prevented the production of pro-inflammatory cytokines in MI

**Fig. 6:**
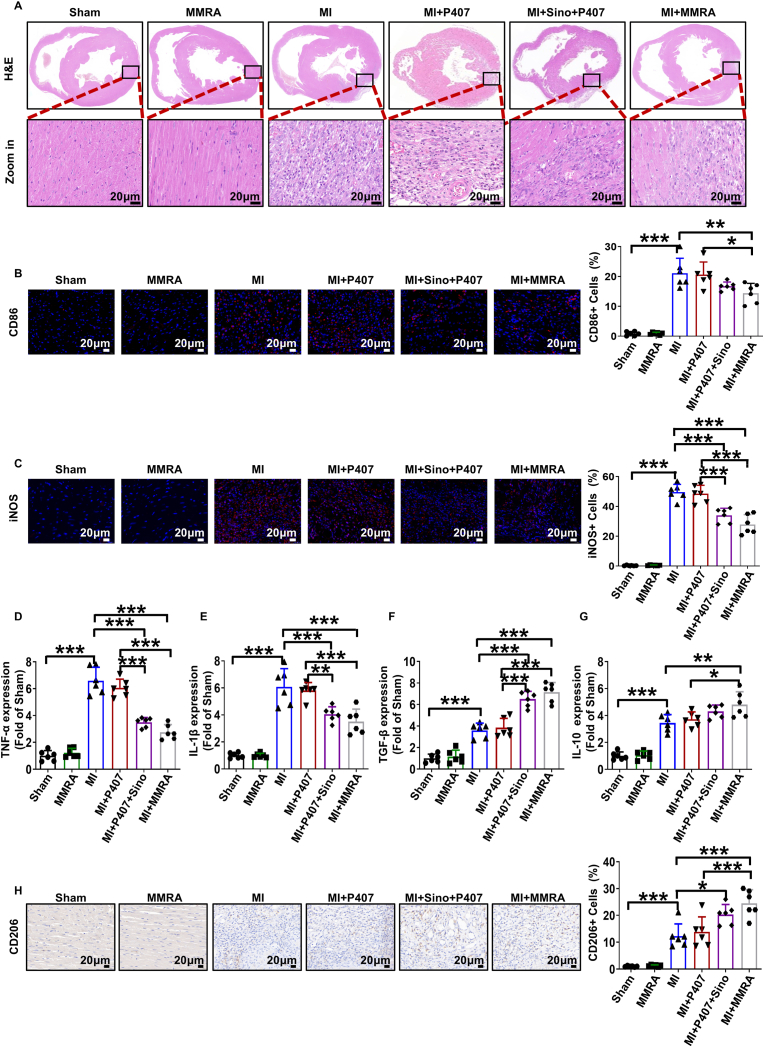
MMRA regulates cardiomyocyte inflammation in MI. (A) Representative images of H&E staining. (B–C) Representative immunofluorescence images and quantification of CD68 and iNOS (n = 6). (D–G) qPCR analysis of the levels of cytokines, including TNF-α, IL-1β, TGF-β and IL-10 (n = 6). (H) Representative immunohistochemical staining and quantification of CD206 (n = 6). ∗*P* < 0.05, ∗∗*P* < 0.01, ∗∗∗*P* < 0.001.

Excessive ROS induces the infiltration of inflammatory cells, creating a vicious cycle of inflammation and ROS production in MI. Inflammation and fibrosis within MI tissue are closely associated with macrophage infiltration and polarization [53]. Macrophage phenotypic shift from M1 to M2 plays a critical role in regulating cardiomyocyte inflammation and promoting tissue repair [12]. The ideal process for tissue repair after MI is the polarization of macrophages from the pro-inflammatory M1 phenotype to the restorative or anti-inflammatory M2, which can reduce chronic inflammation and myocardial fibrosis [16]. Whether MMRA promotes myocardial repair after MI through their anti-inflammatory effects was further evaluated. By H&E staining, we found that a large number of neutrophils were observed in the MI group, along with obvious cell swelling. The MI + Sino + P407 group could improve this situation, while MI + MMRA could significantly alleviate this phenomenon in the tissues of the MI + MMRA group (Fig. 6A). M1 macrophages were stained with CD86 antibodies, indicating that the CD86 levels were significantly elevated in the MI group compared to the Sham group, while being markedly reduced in the MI + MMRA group (Fig. 6B). The expression of key cytokines associated with M1 macrophage polarization, including iNOS, TNF-α, IL-1β, and IL-6, within the infarct zone was significantly reduced in the MI + MMRA group compared with the MI, MI + P407, and MI + Sino + P407 groups (Fig. 6C–E and Fig. S7A). Conversely, the MI + MMRA group exhibited markedly elevated expression of M2 polarized cytokines, such as TGF-β, IL-10, CD206, and Arg-1, relative to the other treatment groups (Fig. 6F–H and Fig. S7B). These results indicate that MMRA may have anti-inflammatory properties, inhibit M1 macrophage polarization, promote M2 polarization, create a favorable environment for myocardial cells, reduce cell apoptosis, delay the loss of functional myocardial cells, reverse left ventricle remodeling, and help maintain cardiac structure and function, thereby aiding in MI treatment.

### 2.7 MMRA improved pathological ventricular remodeling after MI with excellent biocompatibility

**Fig. 7:**
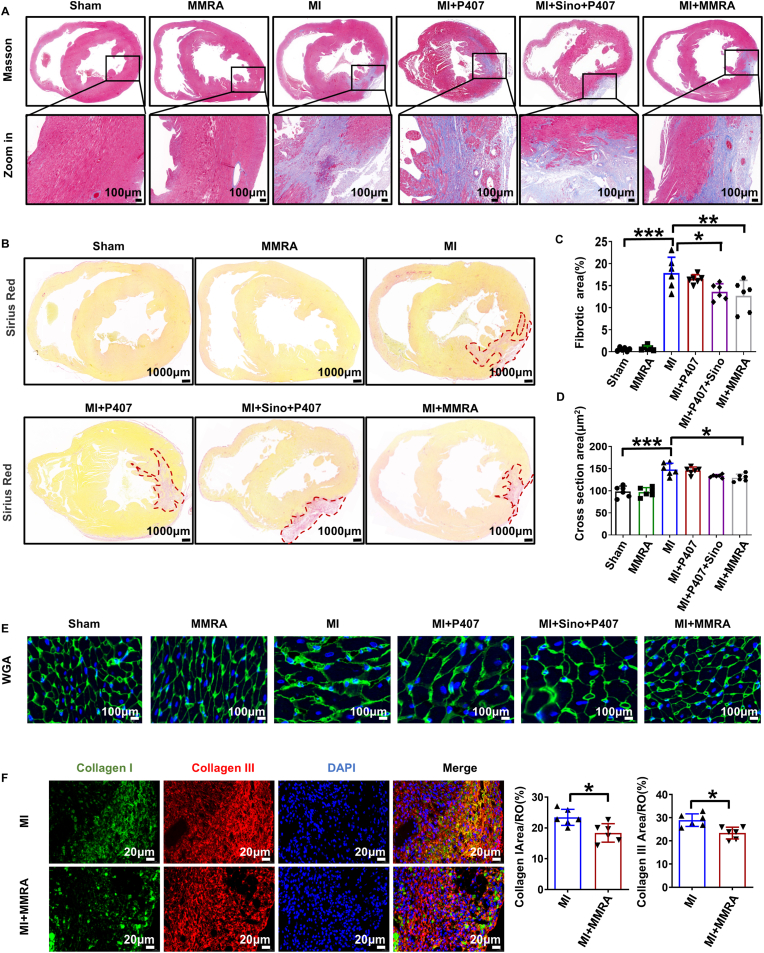
MMRA inhibits myocardial fibrosis After MI. (A) Representative Masson's trichrome staining images. (B) Representative images of Sirius Red staining. (C) Masson's trichrome staining quantitative analysis (n = 6). (D–E) Representative WGA immunofluorescence staining images and quantitative analysis of cardiomyocyte cross-sectional area (n = 6). (F) Representative immunofluorescence staining images of Collagen I and III and quantitative analysis (n = 6). ∗*P* < 0.05, ∗∗*P* < 0.01, ∗∗∗*P* < 0.001.

Ventricular remodeling following MI is an important cause of malignant events such as arrhythmia and cardiac insufficiency. Many factors are involved in ventricular remodeling after MI, including cardiomyocyte hypertrophy, apoptosis, and myocardial fibrosis, especially myocardial fibrosis caused by excessive deposition of Extra Cellular Matrix proteins in the myocardium [54,55]. To determine if the cardioprotective effects of MMRA are linked to reduced cardiac fibrosis, we evaluated the extent of cardiac fibrosis post-MI in mice. Masson and Sirius Red staining revealed a significant increase in myocardial fibrosis within the infarct and border zones in the MI group compared with the Sham group. Among all treatment groups, including MI + P407 and MI + Sino + P407, the MI + MMRA group exhibited the most pronounced attenuation of fibrosis, as evidenced by reduced collagen deposition in both staining methods (Fig. 7A–C). Wheat germ agglutinin (WGA) staining further indicated that MI induced significant cardiomyocyte hypertrophy, an effect that was most markedly suppressed in the MI + MMRA group compared to all other experimental groups (Fig. 7D–E). Furthermore, MMRA treatment markedly prevented the pathological accumulation of collagen I and III in myocardial tissues post-MI (Fig. 7F). These findings suggest that MMRA therapy effectively inhibits myocardial fibrosis following MI and mitigates adverse ventricular remodeling.

Although Sino and P407 are clinically approved materials, the toxicity of PPy degradation products has not been evaluated. This study conducted a comprehensive biosafety assessment of the intact MMRA hydrogel and its individual constituents. The major organs such as the liver, lungs, kidneys, and spleen were excised for histological analysis seven days post-implantation (Fig. S8). H&E staining revealed no observable abnormalities in tissue architecture across all treatment groups. Specifically, examinations showed absent signs of inflammatory cell infiltration, negligible tissue necrosis, and preserved cytoarchitectural organization. All evaluated organs maintained structural integrity, with histological features comparable to those of shame-operated controls. These results indicate that the MMRA hydrogel and its components exhibit favorable biocompatibility under the described experimental conditions, with no evidence of systemic toxicity.

### 2.8 Transcriptome profiling of myocardial tissue

**Fig. 8:**
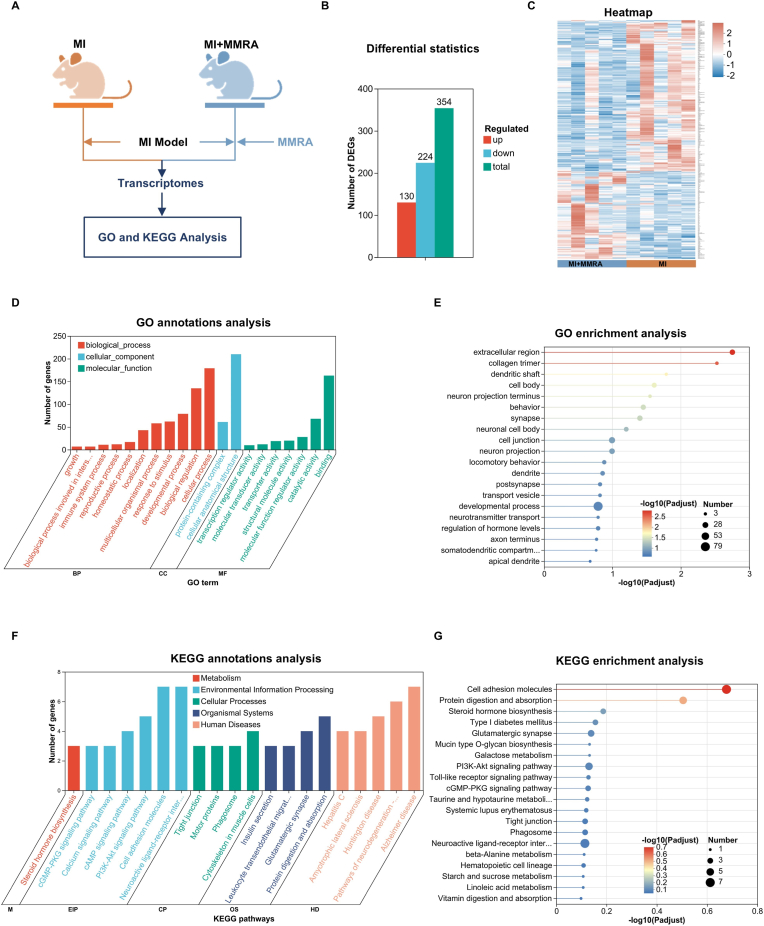
Transcriptomic and Bioinformatics Analysis of MMRA in MI. (A) Schematic diagram of the transcriptomic animal treatment protocol. (B) DEGs statistics of between the MI and the MI + MMRA group. (n = 5). (C) Heatmap. (n = 5). (D–E) GO annotations and enrichment analysis. (n = 5). (E–F) KEGG annotations and enrichment analysis. (n = 5).

To investigate the molecular mechanisms by which MMRA exerts cardioprotective effects after MI, transcriptomic analysis was performed on myocardial tissues from the MI group and the MI + MMRA treatment group (Fig. 8A). RNA sequencing identified a total of 354 differentially expressed genes (DEGs), including 130 upregulated and 224 downregulated genes (Fig. 8B). The expression patterns of these DEGs were visualized using a heatmap (Fig. 8C), which showed clear clustering separation between the two groups, indicating that MMRA treatment markedly altered the transcriptional profile of myocardial tissue. Subsequently, Gene Ontology (GO) and Kyoto Encyclopedia of Genes and Genomes (KEGG) analyses were conducted to further elucidate the biological processes and signaling pathways involved in MMRA-mediated cardiac repair. GO analysis revealed that, in the biological process (BP) category, most DEGs were involved in cellular process, biological regulation, and response to stimulus. In the cellular component (CC) category, they were mainly localized to the extracellular region and collagen trimer, while in the molecular function (MF) category, binding and catalytic activity were predominant (Fig. 8D). Further GO enrichment analysis (Fig. 8E) showed that DEGs were significantly enriched in extracellular region, collagen trimer, and cell body, suggesting that MMRA treatment may promote extracellular matrix remodeling and cellular structural recovery to improve tissue repair after myocardial infarction. To reveal the hierarchical relationships among the enriched terms, a GO directed acyclic graph was constructed (Fig. S9). The results showed that MMRA-regulated genes were mainly concentrated in nodes related to extracellular structure and cell junctions, further supporting its potential role in matrix remodeling and intercellular coupling regulation.

In the MMRA system, P407 serves as a conductive, thermosensitive, and injectable hydrogel matrix that provides a stable local drug release platform, mechanical stabilization of the infarct border zone, and the transmission of electrical signals between cells [56,57]. In addition, PPy can promote the activation of repair functions in electroresponsive tissues such as bone, nerve, and cardiac tissue under electrical stimulation [43,49]. In the transcriptomic analysis, DEGs were enriched in terms such as cell junction, synapse, and ion transport, which are consistent with the effects of P407 and PPy mentioned above, suggesting that MMRA may improve intercellular communication and membrane potential stability during myocardial repair, thereby promoting electrical signal integration and reducing post-infarction electrical remodeling.

KEGG functional classification showed that the DEGs were mainly involved in pathways related to metabolism, environmental information processing, cellular processes, and organismal systems (Fig. 8F). Further KEGG enrichment analysis (Fig. 8G) revealed significant enrichment in multiple pathways closely associated with myocardial protection, intercellular signaling, and energy metabolism, including the PI3K-Akt, cGMP-PKG, calcium signaling, and cell adhesion molecule (CAMs) pathways. Notably, these pathways are highly consistent with the reported cardiovascular protective mechanisms of Sino. A recent review reported that Sino exerts anti-inflammatory, antioxidant, and cytoprotective effects by regulating multiple signaling pathways, including PI3K/Akt, suggesting that Sino within the MMRA system may participate in the regulation of these molecular signaling cascades [58].

In addition, enrichment was also observed in several metabolism- and endocrine-related pathways, such as steroid hormone biosynthesis, galactose metabolism, and protein digestion and absorption, indicating that MMRA may promote cardiac repair by modulating energy metabolism, hormonal balance, and extracellular matrix interactions. The KEGG chord plot (Fig. S10) further showed that several key genes (e.g., Col1a1, Irs2, Cntn2) were simultaneously involved in multiple signaling pathways, exhibiting a multitarget regulatory pattern primarily associated with cell survival, angiogenesis, and energy metabolism.

In summary, the differential gene expression and pathway enrichment results demonstrate that MMRA treatment significantly modulated multiple biological processes, including extracellular matrix remodeling, cell adhesion, oxidative stress response, and energy metabolism. These transcriptional alterations are highly consistent with the coordinated effects of MMRA in regulating inflammation, redox homeostasis, and electromechanical synchronization during myocardial repair.

## 3 Conclusion

In this study, we developed an injectable conductive hydrogel, termed the MMRA, formulated with Sino, PPy and P407. The MMRA shows significant antioxidant and anti-inflammatory properties, as evidenced by its capacity to scavenge ROS, maintain mitochondrial integrity, inhibit M1 macrophage polarization, and promote M2 polarization. Additionally, it enhances electrical integration, facilitating synchronized cardiomyocyte contraction and gap junction communication. After the injection of MMRA in mice with MI, cardiac function was significantly restored due to its effect of improving the myocardial microenvironment, showing that cardiomyocyte apoptosis and infarct size decreased, fibrosis degree improved, and left ventricular ejection fraction increased. These findings highlight the translational potential of MMRA in the clinical application of MI treatment.

## 4 Experimental section

Detailed information regarding the synthesis and characterization of MMRA, as well as relevant experimental details for in vitro and *in vivo* studies of MMRA, are provided in Supporting Information.

## CRediT authorship contribution statement

**Kaiyi Zhu:** Writing – review & editing, Supervision, Resources, Funding acquisition, Formal analysis, Conceptualization. **Xiaozhe Wang:** Writing – review & editing, Writing – original draft, Validation, Methodology, Investigation, Data curation. **Qian Yang:** Writing – review & editing, Writing – original draft, Visualization, Methodology, Investigation, Data curation. **Qiuyi Liao:** Methodology, Investigation. **Sunli Liu:** Writing – original draft, Methodology, Investigation. **Yuchen Cao:** Methodology, Investigation. **Wei Yang:** Resources, Investigation, Conceptualization. **Xueyan Li:** Methodology, Formal analysis, Conceptualization. **Xiaolong Mi:** Validation, Resources. **Yuanyuan Lin:** Validation, Supervision, Conceptualization. **Qiang Zhou:** Conceptualization. **Yue Song:** Supervision. **Chunbo Dong:** Writing – review & editing. **Yuping Gao:** Validation, Supervision, Project administration, Funding acquisition. **Zhida Liu:** Writing – review & editing, Supervision, Resources. **Liping Li:** Writing – review & editing, Supervision, Resources, Project administration, Funding acquisition, Data curation. **Ruiping Zhang:** Supervision, Resources, Project administration, Funding acquisition.

## Funding

This work was financially supported by the Basic Research Program of Shanxi Province (202203021222340), 10.13039/501100001809National Natural Science Foundation of China (82120108016, 82071987, U22A20349, and 82001962), the Central Government Guided Local Science and 10.13039/100006180Technology Development Fund Research Project (YDZJSX20231A055), the National Key R&D Program of China (2023YFC3402800), Shanxi Traditional Chinese Medicine Administration research topic (2024ZYYC032), Scientific research start-up fund project for talent introduction of Shanxi Bethune Hospital (2023RC23), and the Key Laboratory of Nano-imaging and Drug-loaded Preparation of Shanxi Province (202104010910010).

## Declaration of competing interest

The authors declare that they have no known competing financial interests or personal relationships that could have appeared to influence the work reported in this paper.

## Data Availability

Data will be made available on request.
