# Supplementary material for: A tripartite therapeutic agent reprograms the myocardial infarction microenvironment
Source: Mater Today Bio. 2025 Nov 24;35:102600. doi: 10.1016/j.mtbio.2025.102600 (PMC12702353; doi:10.1016/j.mtbio.2025.102600)
Supplement: Multimedia component 1 [file mmc1.docx]

**A Tripartite Therapeutic Agent Reprograms the Myocardial Infarction Microenvironment**

Kaiyi Zhu^a1^, Xiaozhe Wang^f1^, Qian Yang^a1^, Qiuyi Liao^a^, Sunli Liu^a^, Yuchen Cao^a^, Wei Yang^b^, Xueyan Li^c^, Xiaolong Mi^a^, Yuanyuan Lin^a^, Qiang Zhou^a^, Yue Song^a^, Chunbo Dong^d^, Yuping Gao^a^*, Zhida Liu^d,f^*, Liping Li^e^*, Ruiping Zhang^f^*

Experimental Section

*Materials:* All aqueous solutions were prepared using ultrapure water. Pyrrole (py) solution, ammonium persulfate (APS), and β-cyclodextrin (β-CD) were provided by Energy Chemicals (Shanghai, China). Planctinic 407, Superoxide anion assay kit, total antioxidant capacity assay kit (ABTS rapid method) and DPPH Free Radical Scavenging Capacity Assay Kit were obtained from Solarbio Science & Technology Co Ltd. (Beijing, China). All reagents were not further purified. H9C2 and RAW264.7 were supplied by the MOH Key Laboratory of Geriatrics (Beijing Hospital). High glucose dulbecco’s modified Eagle’s medium (DMEM), fetal bovine serum (FBS) and Penicillin-Streptomycin were purchased from HyClone (USA). Hydrogen peroxide (H₂O₂, 30% solution) and lipopolysaccharide (LPS) were purchased from Beijing Chemical Industry Co., China. The PBS(HT112) was obtained from Shaanxi ZHHC Biopharmaceutical Technology Co., Ltd. The Live/Dead cell double staining kit (L32250) sourced from Thermo Fisher Scientific (USA). Anti-CD86 (ab119857), anti-iNOS (ab178945), anti-Cx-43 (ab11370), anti-α-actinin (ab68194), anti-Collagen I (ab138492), anti-Collagen III (ab184993) and Trichrome Stain Kit (ab150686) were purchased from Abcam (USA). Anti-CD206 (#24595) was purchased from CST (USA). TUNEL staining kit (Apo Alert DNA Fragmentation Assay kit, Clontech) was provided by BD Biosciences (USA). HE Stain Kit (G1120), 3-(4,5-dimethylthiazol-2-yl)-2,5-diphenyltetrazolium bromide (MTT) assay kit (M8180) and 2,3,5-Triphenyl-2H-tetrazolium chloride (TTC, T8170) were purchased from Solarbio Science & Technology Co Ltd. (Beijing, China). TRIzol reagent kit and reverse transcription system were provided by Invitrogen, Thermo Fisher Scientific (USA). SYBR q-PCR kit was purchased from TAKARA (Japan). The enzyme-linked immunosorbent assay (ELISA) test kits of

IL-10, IL-1β, TGF-β and TNF-α were purchased from Winter Song Boye Biotechnology Co. Ltd. (Beijing, China). Flow cytometry reagent (B358391) was purchased from BioLegend (USA). QIAzol Lysis Reagent was purchased from Qiagen (Hilden, Germany). RNA Purification Kit was purchased from Shanghai Meggie Bioscience Co., Ltd. (Shanghai, China). SMART-Seq® v4 Ultra Low Input RNA Kit was purchased from Takara Bio USA, Inc. (Mountain View, CA, USA).

*Synthesis and Characterization of Ppy:* Add 0.5 g β-CD and 15 mL of ultrapure water in a beaker, stir until β-CD completely dissolved and then add 69 µL pyrrole. After stirring for 3 h, 176 mg of APS was added and reacted for 12 h. Centrifugation was performed, and the precipitate was washed twice with ultrapure water and once with anhydrous ethanol, and the precipitate was separated and dried under vacuum at 50 ℃ for 24 h. The complex PPy of β-cyclodextrin and polypyrrole was obtained, and the product was a black solid. The synthesis of PPy was confirmed using a UV-Vis-NIR spectrophotometer (HL-2000, TS OPTICS, China) and a Fourier transform infrared spectrometer (Vertex Perkin-Elmer 580BIR, Bruker, America). The morphology of PPy was observed using a projection electron microscope (JEM-2100F, JEOL, Japan) and a scanning electron microscope (JSM-6510, JEOL, Japan). The morphology of MMRA was observed using a scanning electron microscope (G4UC, Helios, USA).

*Synthesis and Characterization of MMRA:* The Sino and PPy were mixed with P407 solution at a concentration of 20% w/v and then stirred at 4 ℃ for 10 min to ensure that Sino and PPy were uniformly distributed in the P407 solution, after obtaining MMRA with a Sino and PPy concentration of 50 μg/mL.

The properties of temperature-sensitive hydrogels were evaluated using the tilt method and rheological tests. P407 or MMRA solutions obtained at 4 ℃ were placed in a water bath at 37 ℃, and gelatinization was determined by observing the absence of liquid flow after tilting.

The thermosensitization process of hydrogels was tested using an advanced rotational rheometer (MCR102, Anton Paar, Austria), where the storage modulus (G′) and loss modulus (G″) of hydrogels were measured by increasing the temperature from 0 ℃ to 45 ℃. The frequency was increased from 1 rad/s to 100 rad/s at 37℃ to measure hydrogels G′ and G″. The hydrogels G′ and G″ of the hydrogels at 37 ℃ constant frequency of 1 Hz at 0-300 s conditions.

Four-probe Resistivity Test, electrical resistivity of the samples was measured using an AC four-point probe resistivity tester (ST2242) with a thin-film probe head (ST2558B-F01). Measurements were performed on three times on each sample at room temperature. Resistivity (Ω·m) was recorded directly, and conductivity (S/cm) was calculated as the reciprocal of resistivity according to the instrument formula.

The antioxidant properties of Sino, PPy aqueous solution and MMRA were tested using SOD activity assay kit, DPPH free radical scavenging capacity assay kit and ABTS free radical scavenging capacity assay kit, respectively, in which the concentration of Sino and PPy aqueous solution was 50 μg/mL, and all the operations were performed according to the instructions.

*Cell Culture:* H9C2 rat cardiomyocytes and RAW264.7 cells were routinely cultured in high glucose DMEM supplemented with 10% heat-inactivated fetal bovine serum and 1% penicillin-streptomycin at 37 ℃ in a 5% CO_2_ incubator. Cells were passaged when they reached 80% confluence, and cells in the logarithmic growth phase were used for experiments.

H9C2 cells were treated with H2O2 (200 μM) at 37℃ for 24 hours to simulate ROS-induced myocardial cell damage in vitro. H9C2 cells were co-incubated with H2O2 or varying concentrations of MMRA for a duration of 24 hours, which was used to assess the role of MMRA in the oxidative stress environment. RAW264.7 cells were induced using LPS (100 ng/mL) to simulate the inflammatory state of macrophage. RAW264.7 cells were co-incubated with or without hydrogel of MMRA for 24 hours to evaluate the effect on macrophage polarization in vitro.

*Cell Viability Assay:* Incubate the cells with different concentrations of MMRA in a 96-well plate for 24 hours. Following MMRA removal, cell viability was evaluated using an MTT assay kit in accordance with the manufacturer's instructions. Finally, the optical density (OD value) was measured at a wavelength of 490 nm. Cell viability (%) was calculated as (OD value of the experimental sample/OD value of the control group) × 100%.

*LDH Release Assessment:* Incubate the cells with different concentrations of MMRA in a 96-well plate for 24 hours. After removing the drug, following the manufacturer's protocol, an LDH assay kit was utilized to determine the LDH levels in the culture medium of H9C2 cells using an enzyme-linked immunosorbent assay ELISA.

*Flow cytometry:* RAW264.7 cells were seeded into 6-well plates and stimulated with LPS. The experimental group received MMRA treatment. After 24 h, cells were harvested and incubated with an anti-CD86 antibody for surface staining. Cells were then fixed and permeabilized using a commercial fixation/permeabilization kit and incubated with an anti-CD206 antibody. Finally, stained cells were analyzed by flow cytometry.

*ELISA:* Culture supernatants were collected from the 6-well plates described above immediately before cell harvest and centrifuged at 300 × g for 20 min to remove cellular debris. Secretion levels of IL-10, IL-1β, TGF-β and TNF-α were measured using commercial ELISA kits according to the manufacturers’ instructions. Samples and standards were assayed following the kit protocols.

*Live/Dead Staining:* H9C2 cells were plated in 96-well plates, and the Live/Dead Cell Double Staining Kit was used according to the manufacturer’s instructions. Fluorescent images were acquired using a microscope (Olympus, Tokyo, Japan), and cell quantification was conducted with ImageJ software.

*Detection of Oxidative and Antioxidant Biomarkers:* The content of malondialdehyde (MDA) was measured at 532 nm using a thiobarbituric acid assay kit (Beyotime). Glutathione (GSH) levels were determined at 412 nm following the manufacturer's instructions for the reduced GSH assay kit (Solarbio). Catalase (CAT) activity was calculated based on the rate of absorbance reduction at 240 nm. Total superoxide dismutase (T-SOD) activity was assessed using the hydroxylamine method, measuring absorbance at 560 nm according to the instructions provided with the T-SOD assay kit (Solarbio).

*Mouse Model of MI:* All animal experiments were conducted according to the protocols approved by the Institutional Animal Care and Use Committee of the Animal Experiment Center of Shanxi Medical University (No.2016LL141, Taiyuan, China). C57BL/6 mice were purchased from SPF Biotechnology (Beijing, China). All mice were housed in pathogen-free conditions at the animal care facility of Shanxi Bethune Hospital. They were maintained at room temperature with free access to food and water under a 12-hour light/dark cycle. Throughout the study, all mice used in the experiments exhibited normal health conditions. Mice were used after a one-week acclimation period. All animal studies were conducted in accordance with the Guidelines for the Care and Use of Laboratory Animals (Ministry of Science and Technology of China, 2006).

The experimental subjects were male C57BL/6 mice, aged 8-10 weeks and weighing between 22-25 g. Following mechanical ventilation and anesthesia with pentobarbital (40 mg/kg), a left thoracotomy was performed to expose the heart. The left anterior descending coronary artery (LAD) was permanently ligated approximately 2 mm distal to the tip of the left atrial appendage using 6-0 sutures to establish a MI model in mice. Successful establishment of the MI model was confirmed by observing pallor and ST-segment elevation on electrocardiogram testing. After MI induction, 20 μL of PBS or hydrogels were administered via multiple injections into the border zone (4 points, each point 5 μL). The sham-operated group underwent an identical thoracotomy procedure without coronary artery ligation.

*Echocardiography:* Cardiac function of the left ventricle in all groups of animals was assessed using the Vevo 3100 ultrasound imaging system (Visual Sonics, Canada). Echocardiographic examinations were conducted on 7 days and 28 days of post-treatment. M-mode was used with the short-axis view positioned at the level of the papillary muscles to measure left ventricular ejection fraction (LV-EF%), left ventricular fractional shortening (LV-FS%), left ventricular internal diameter in diastole (LVIDd), and left ventricular internal diameter in systole (LVIDs). The measurements were averaged over three consecutive cardiac cycles.

*Immunohistochemistry:* Hearts from mice were perfused with PBS and subsequently fixed in 4% paraformaldehyde overnight at 4℃, followed by embedding in paraffin. The heart tissue was then sectioned into 5μm thick slices for further staining procedures. The slices were incubated in a solution of 5% bovine serum albumin and 0.25% Triton X-100, washed with PBS at room temperature, and then incubated overnight at 4℃ with the following primary antibodies: anti-CD86 (1:200), anti-CX43 (1:400), anti-α-actinin (1:400), anti-Collagen I (1:200) and anti-Collagen III (1:200). Following this incubation step, the slices were treated with a secondary antibody for one hour and stained using diamidino-2-phenylindole (DAPI) at a dilution of 1:1000 for five minutes before being observed under a fluorescence microscope (Olympus Corporation; Japan). Fluorescence intensity was quantified using ImageJ software. For Terminal deoxynucleotidyl transferase dUTP nick-end labeling (TUNEL) staining of apoptotic cells, the slides were stained with a commercial TUNEL staining kit and counterstained with DAPI. For the Masson trichrome and Hematoxylin-Eosin (H&E) staining of cardiac sections, the Trichrome Stain Kit and H&E Stain Kit were utilized separately.

The polarization of macrophages was evaluated by measuring the fluorescence intensity of iNOS and CD206 markers. RAW264.7 cells or 5-micron thick frozen heart tissue sections were stained for iNOS and CD206 following the manufacturer's instructions. Fluorescence images were captured using a fluorescence microscope (Olympus, Tokyo, Japan) and quantified utilizing NIH ImageJ software.

*In Situ Detection of Reactive 0xygen Species (ROS):* The production of intracellular ROS was assessed by measuring the fluorescence intensity of 2,7-dichlorodihydrofluorescein. H9C2 cells or 5-micron thick frozen heart tissue sections were loaded with 10 microns of DAPI and dihydroethidium (DHE) probes and incubated in a dark, humidified environment at 37℃ for 30 minutes. ROS images were captured using a fluorescence microscope (Olympus, Tokyo, Japan) and quantified utilizing NIH ImageJ software.

*TTC Staining:* Infarct areas were assessed by TTC staining. The hearts were first removed, washed three times with precooled saline, and frozen at -80℃ for 5 min. Then, the hearts were dissected into five slices. The slices of hearts were incubated in 2% TTC at 37℃ for 15 min and photographed with a digital camera, to analyze TTC-stained areas (red, normal areas) and non-TTC stained areas (white or pale, infarct areas). Finally, infarct size in the heart was measured using ImageJ software.

*Transmission Electron Microscopy (TEM):* For transmission electron microscopy, heart specimens were cut into 2×2 mm, fixed with 2.5% glutaraldehyde, embedded in epoxy resin, and observed using a transmission electron microscope (Hitachi, Japan).

*Quantitative polymerase chain reaction (qPCR):* Total RNA was extracted from peri-infarct myocardial tissue using the TRIzol reagent kit according to the manufacturer's instructions. Two micrograms of RNA were reverse transcribed into cDNA using the reverse transcription system. Subsequently, the expression levels of pro-inflammatory genes (TNF-α, IL-1β, IL-6) and anti-inflammatory genes (TGF-β, Arg1, IL-10) were measured using primers and SYBR Green dye, following the instructions of the SYBR q-PCR kit (Table 1).

**Table 1 Primer sets used for qPCR**

|  | Forward primer (5′-3′) | Reverse primer (5′-3′) |
| --- | --- | --- |
| IL-6 | AGAGATACAAAGAAATGATGGA | GCTATGGTACTCCACAAGACCA |
| IL-1β | CCAGCAGGTTATCATCATCATCC | CTCGCAGCAGCACATCAAC |
| Arg-1 | GGAAGACAGCAGAGGAGGTG | TATGGTTACCCTCCCGTTGA |
| IL-10 | GGCAGAGAACCATGGCCCAGAA | AATCGATGACAGCGCCTCAGCC |
| β-actin | CCGTGAAAAGATGACCCAGATC | CACAGCCTGATGGCTACGT |
| TNF-α | ATGAGCACAGAAAGCATGATC | TACAGGCTTGTCACTCGAATT |
| TGF-β | CTTCAGCTCCACAGAGAAGAACTGC | CACGATCATGTTGGACAACTGCTCC |

*α-actinin and CX43 Staining:* H9C2 cells were seeded into 96-well plates and subsequently stained for α-actinin and CX43 according to the manufacturer's instructions. Fluorescence images were acquired using an Olympus microscope (Tokyo, Japan) and quantified using ImageJ software.

*Electrocardiography (ECG):* Electrocardiographic recordings were performed in mice using a PowerLab 8/35 system (BL-420N). Mice were lightly anesthetized with 1–2% isoflurane and placed on a heated platform, and subcutaneous needle electrodes were inserted in a lead II configuration. ECG signals were recorded and analyzed using LabChart software.

*Toxicity Assessment:* Fixed liver, kidney, lung, and spleen specimens were dehydrated, cleared, paraffin-embedded, and sectioned at 4 µm. Sections were stained with H&E and imaged by bright-field microscopy.

*RNA Sequencing and Transcriptome Profiling:* RNA was extracted from myocardial tissue using QIAzol Lysis Reagent. Purified RNA was obtained using the RNA Purification Kit. RNA concentration and integrity were assessed with a NanoDrop 2000 spectrophotometer (Thermo Fisher Scientific, USA) and an Agilent 5300 Bioanalyzer (Agilent, USA). For library preparation, samples with sufficient RNA quantity were processed using the Illumina® Stranded mRNA Prep, Ligation Kit (Illumina, USA), while low-input samples were prepared with the SMART-Seq® v4 Ultra Low Input RNA Kit. Libraries were sequenced on the Illumina NovaSeq X Plus platform using NovaSeq Reagent Kits (Illumina, USA). Sample homogenization and pre-processing were performed using a TL-48R tissue grinder (Wanbo Biotechnology, China), ABSON MiFly-6 microcentrifuge (Aibosen, China), and Eppendorf 5424R refrigerated centrifuge (Eppendorf, Germany). Gel electrophoresis was performed using Biowest agarose (Spain). Library quantification was conducted with a Qubit 4.0 fluorometer (Thermo Fisher Scientific, USA), and PCR amplification was performed using a T100 Thermal Cycler (Bio-Rad, USA).The resulting sequencing data were subsequently used for differential gene expression analysis, GO and KEGG functional annotation, and enrichment analysis.

*Statistical Analysis:* The results are presented as mean ± standard deviation (SD). Statistical comparisons were performed using one-way ANOVA followed by unpaired t-tests, utilizing GraphPad Prism 8.0. A p-value below 0.05 (P < 0.05) was considered indicative of statistical significance.


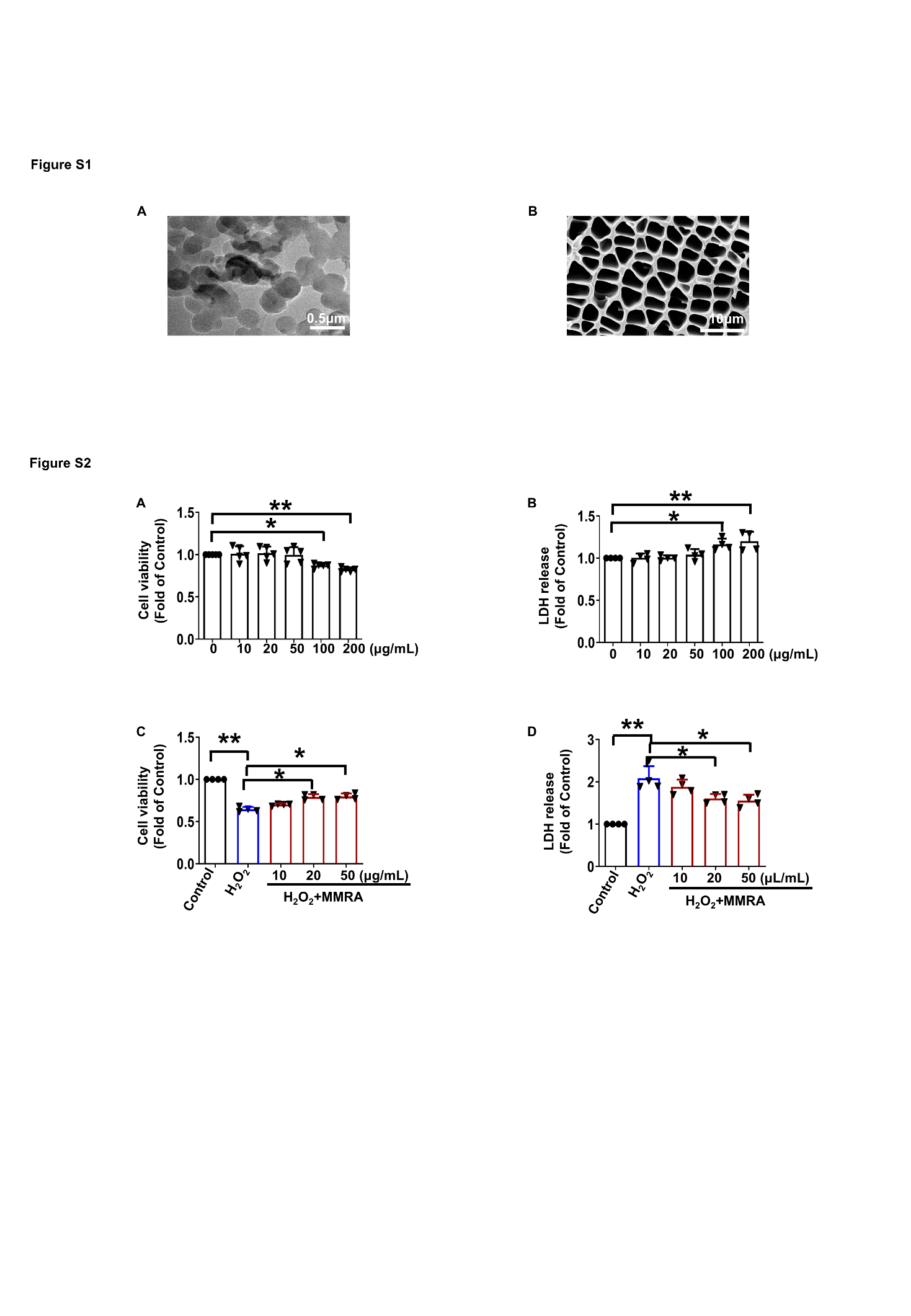


**Fig. S1.** (A) TEM image of PPy. (B) SEM image of P407.


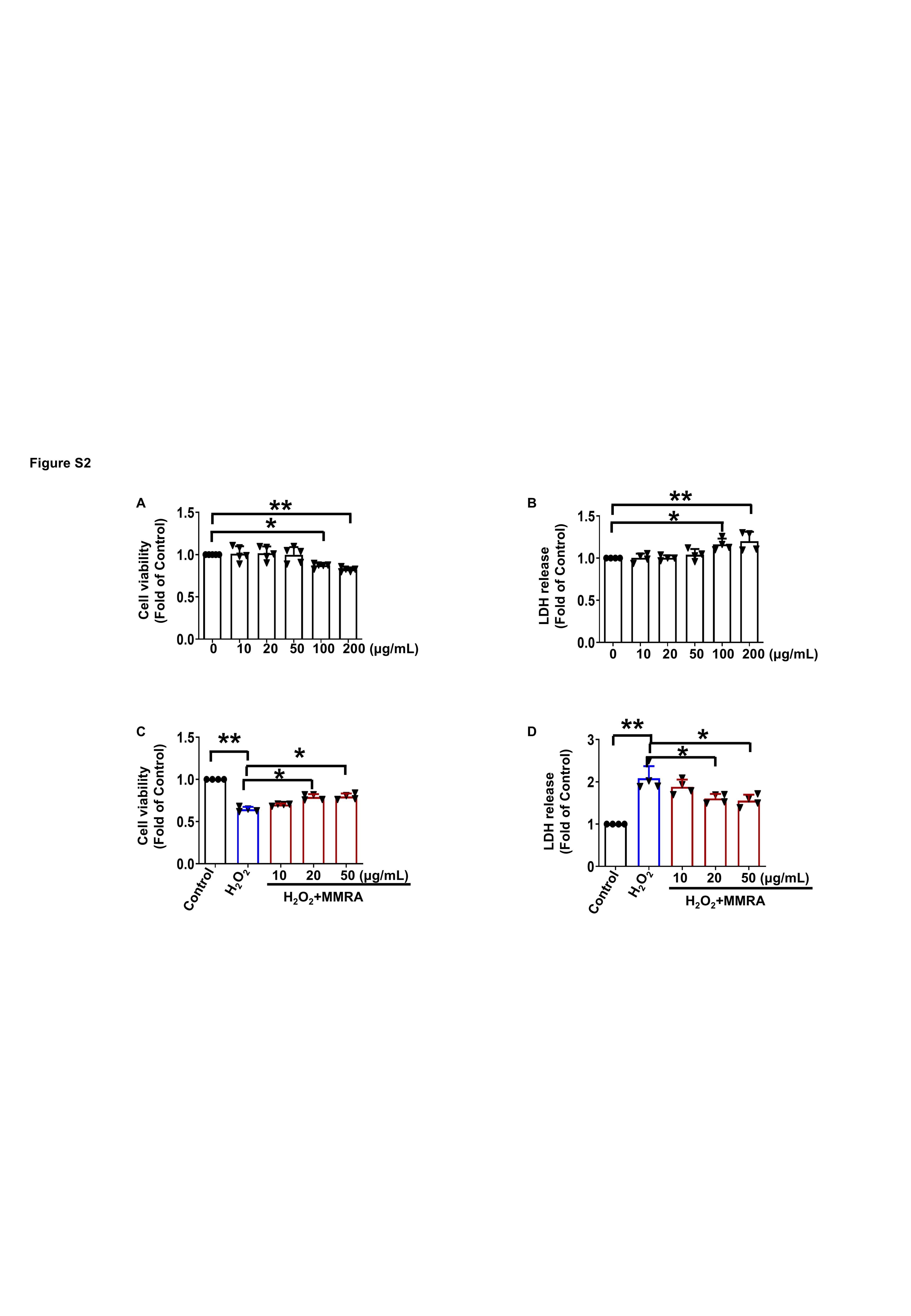


**Fig. S2.** (A-B) Cell viability (n=5) and LDH release (n=4) from H9C2 cells treated with diverse doses of MMRA under normal circumstances. (C-D) Cell viability (n=4) and LDH release (n=4) of H9C2 cells treated with various doses of MMRA under H_2_O_2_-induced oxidative stress (n=4). **P*<0.05, ***P*<0.01.


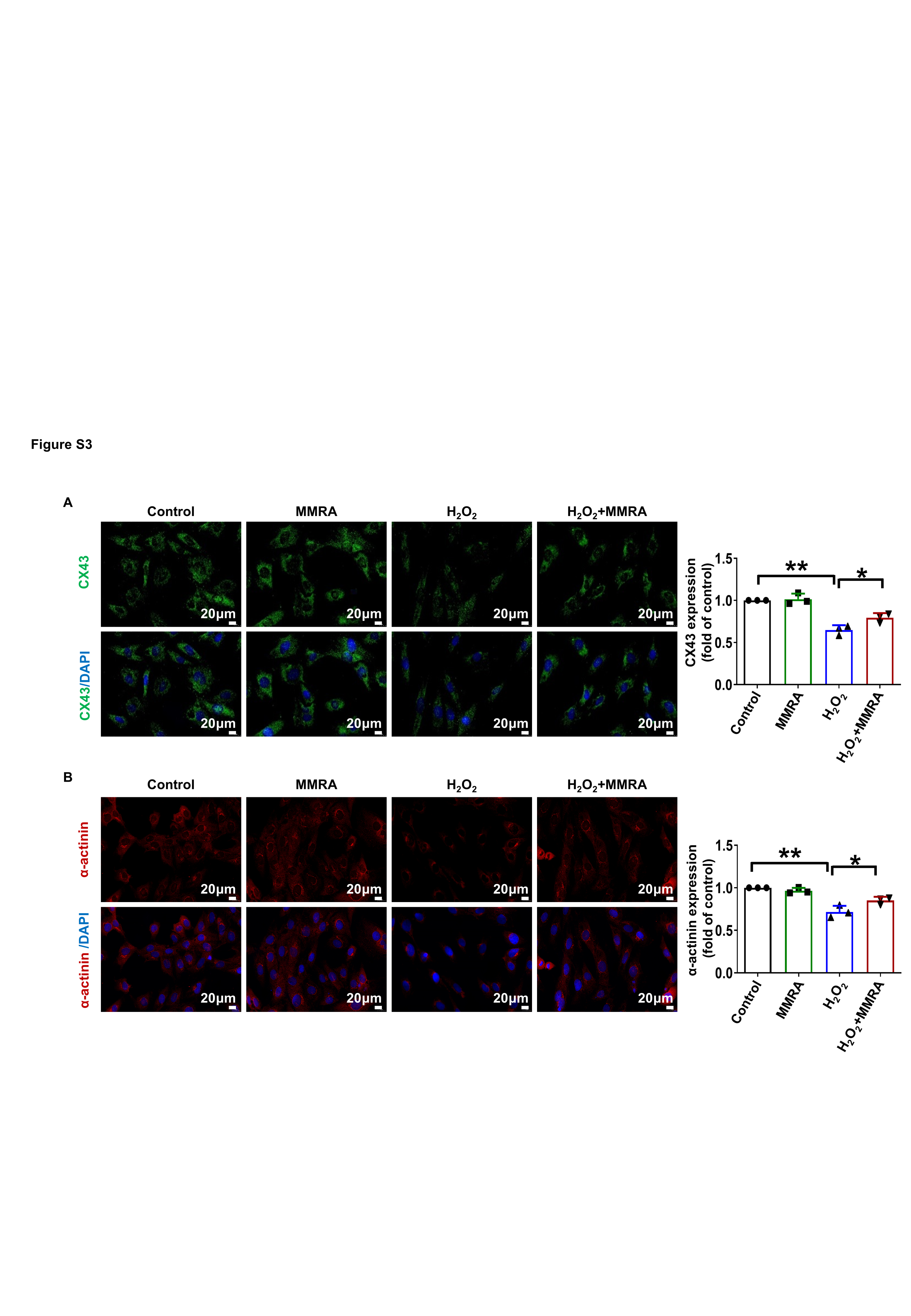


**Fig. S3.** (A-B) Immunofluorescence staining of Cx43 and α-actinin in H9C2 cells (n=3). *p<0.05, ***P*<0.01.


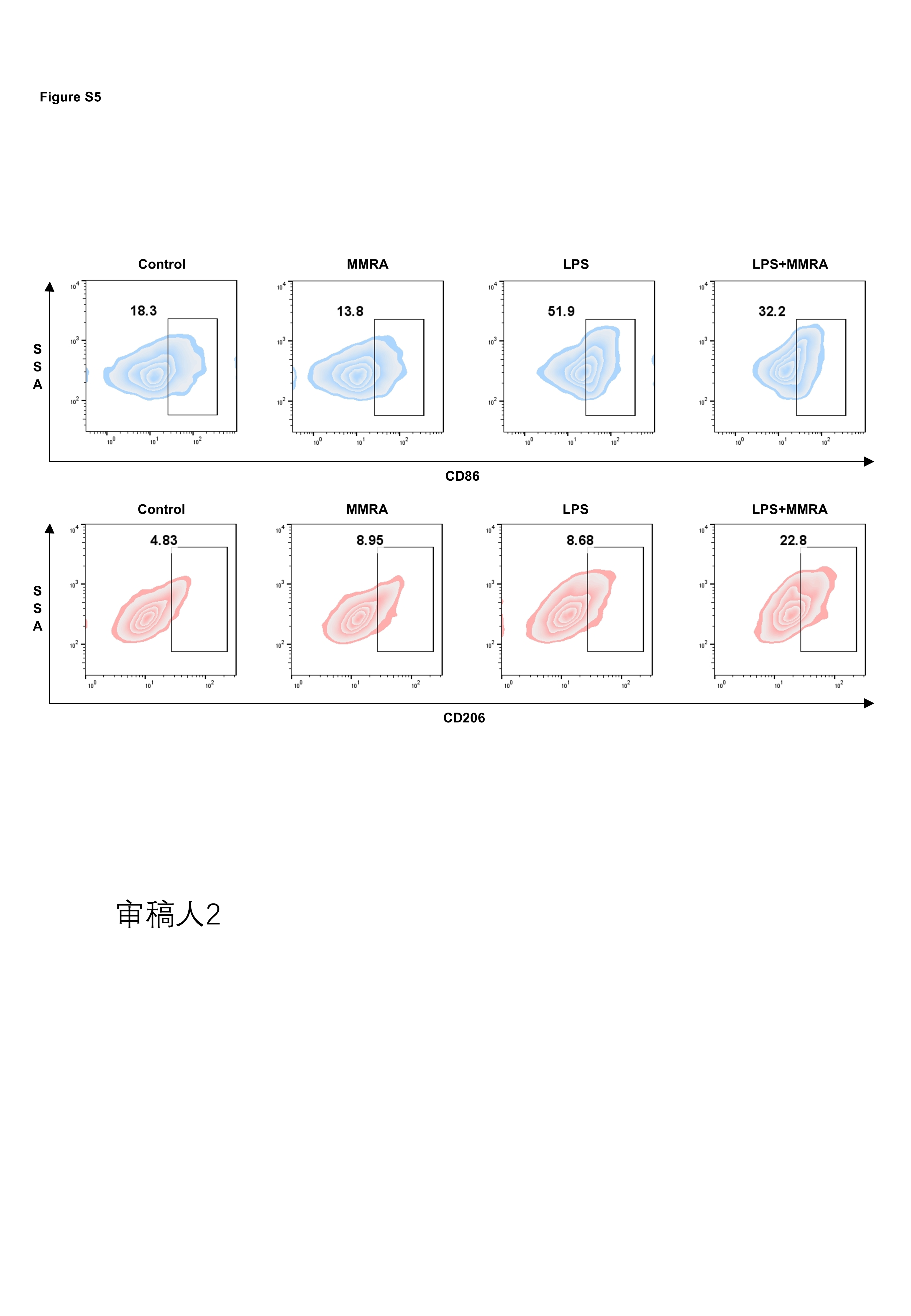


**Fig. S4.** Representative flow cytometry plots of RAW264.7 cells stained with CD86 (M1 marker) and CD206 (M2 marker).


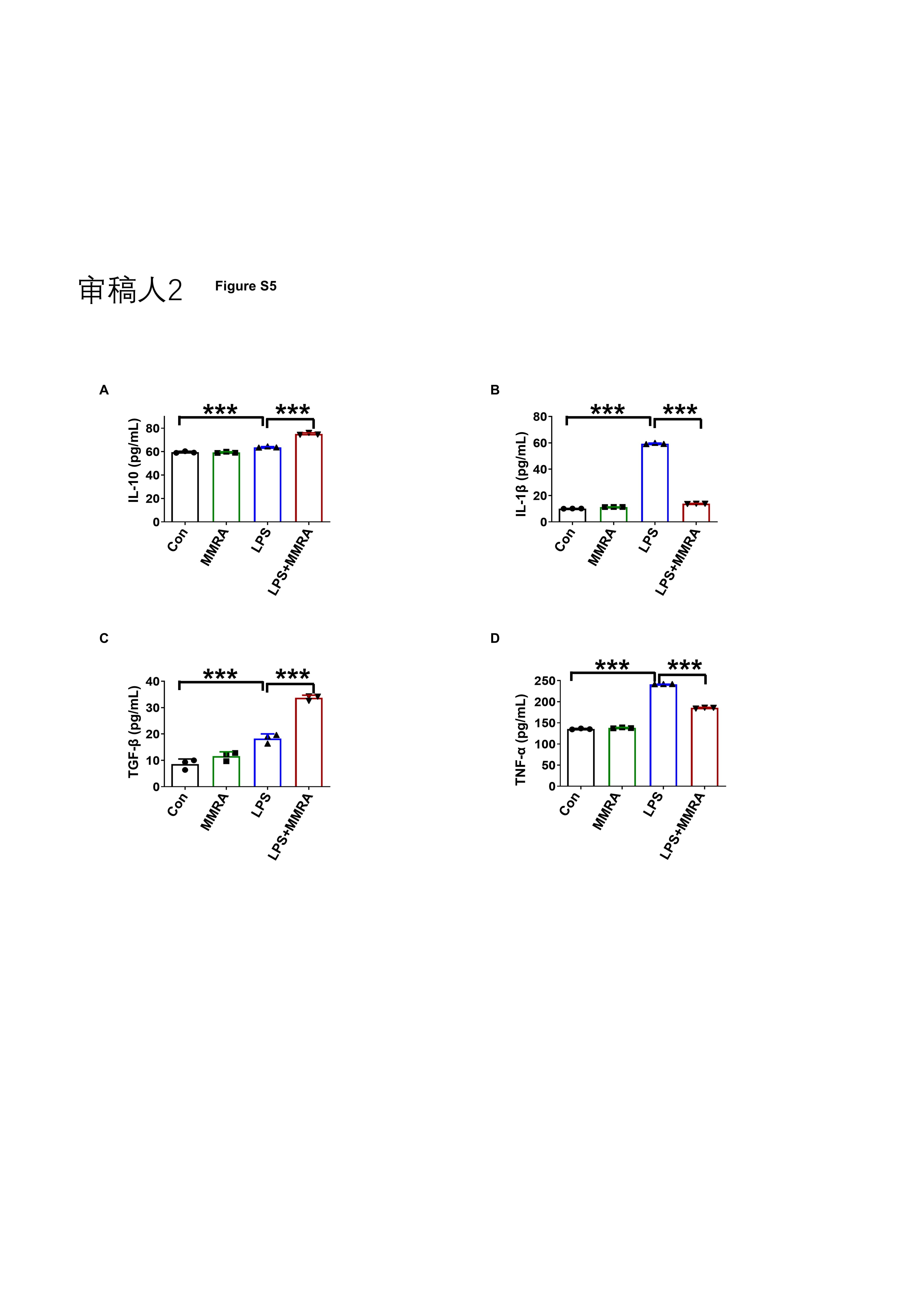


**Fig. S5.** ELISA analysis of cytokine secretion in RAW264.7 cells. Levels of (A) IL-10, (B) IL-1β, (C) TGF-β, and (D) TNF-α were quantified in different groups (n=3). ****P*<0.001.


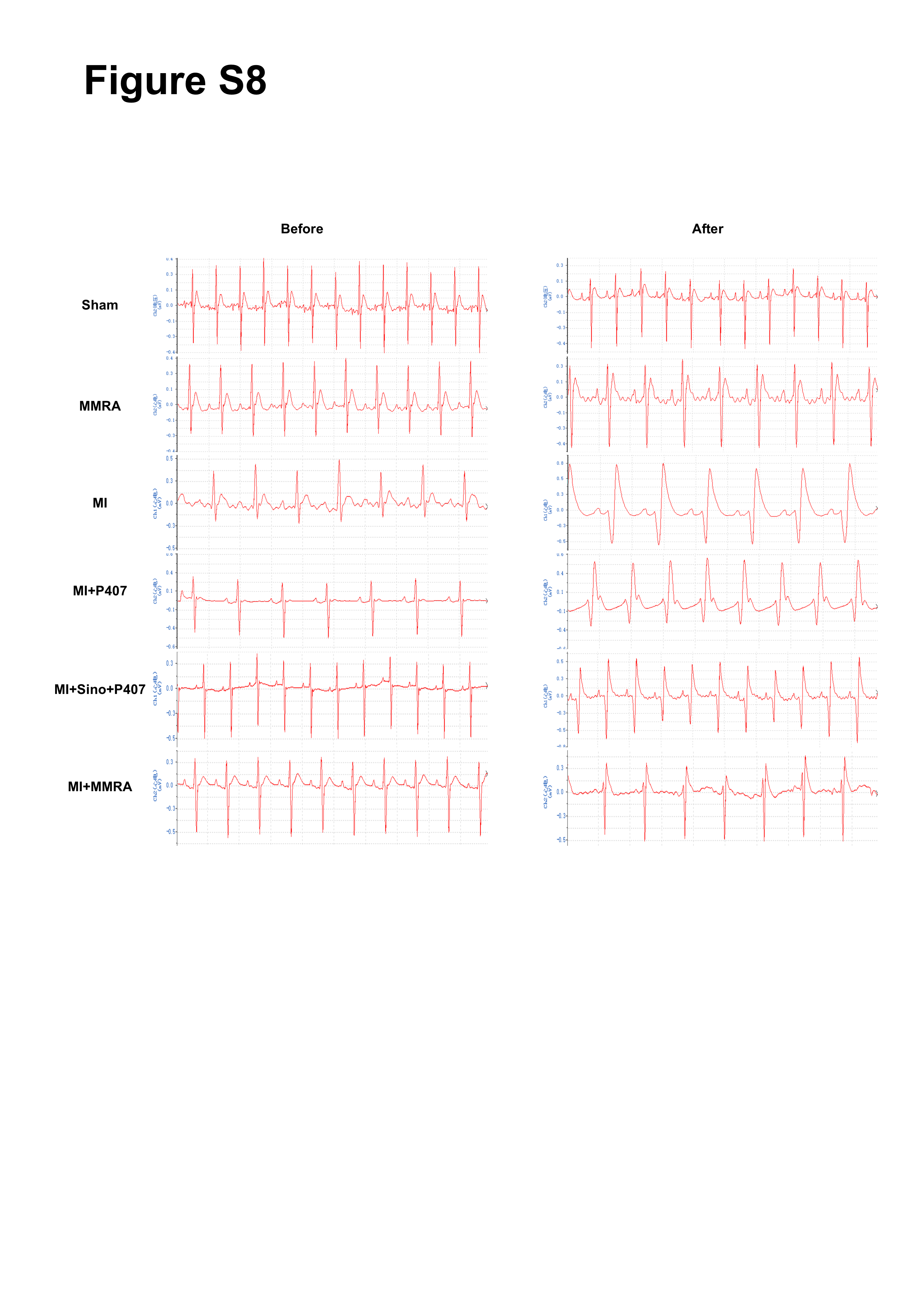


**Fig. S6.** Representative ECG 7 days after MI.


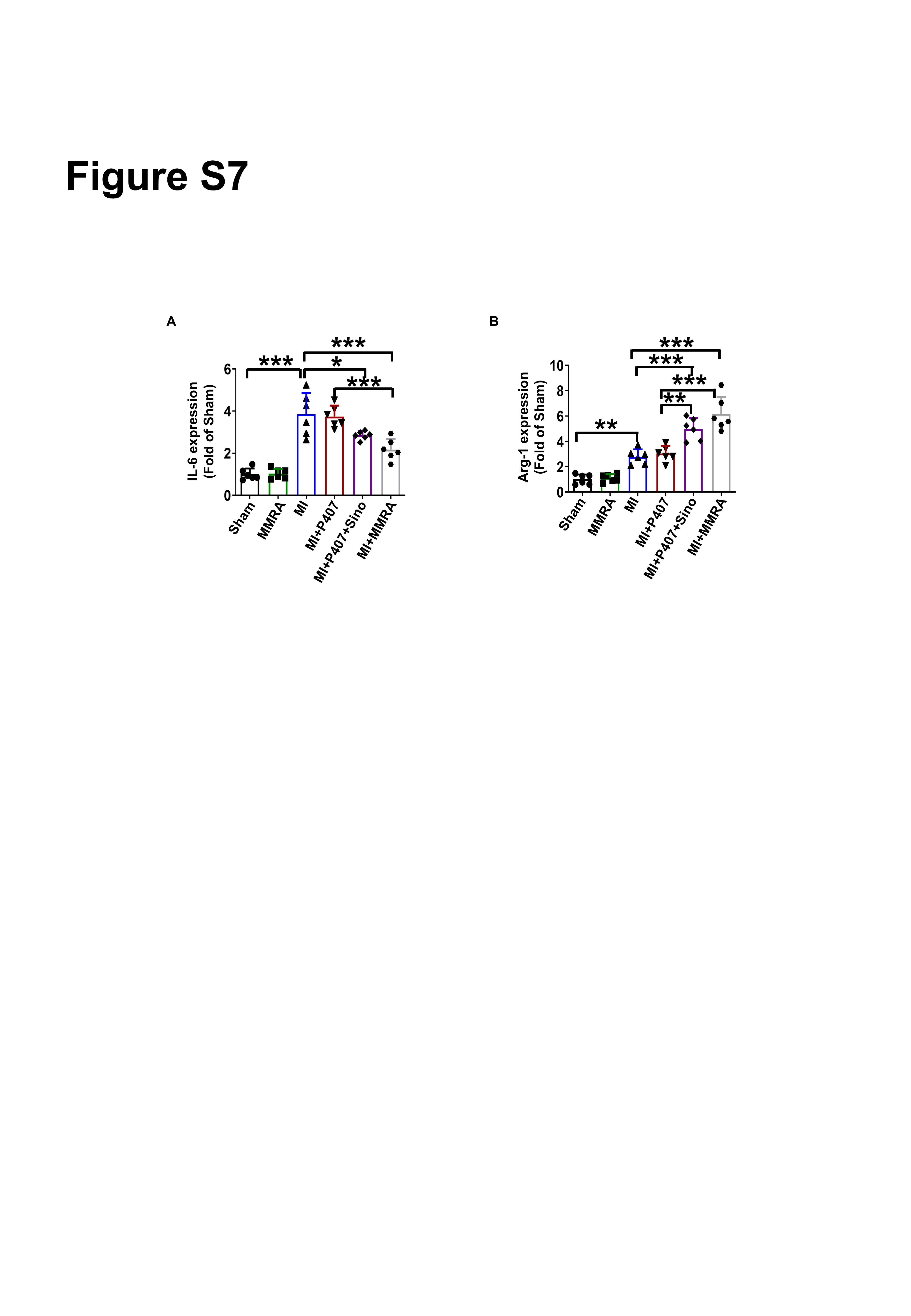


**Fig. S7.** Quantitative PCR analysis of cytokine (A) IL-6 and (B) Arg-1 expression in mouse heart tissues (n=6). *p<0.05, ***P*<0.01, ****P*<0.001.


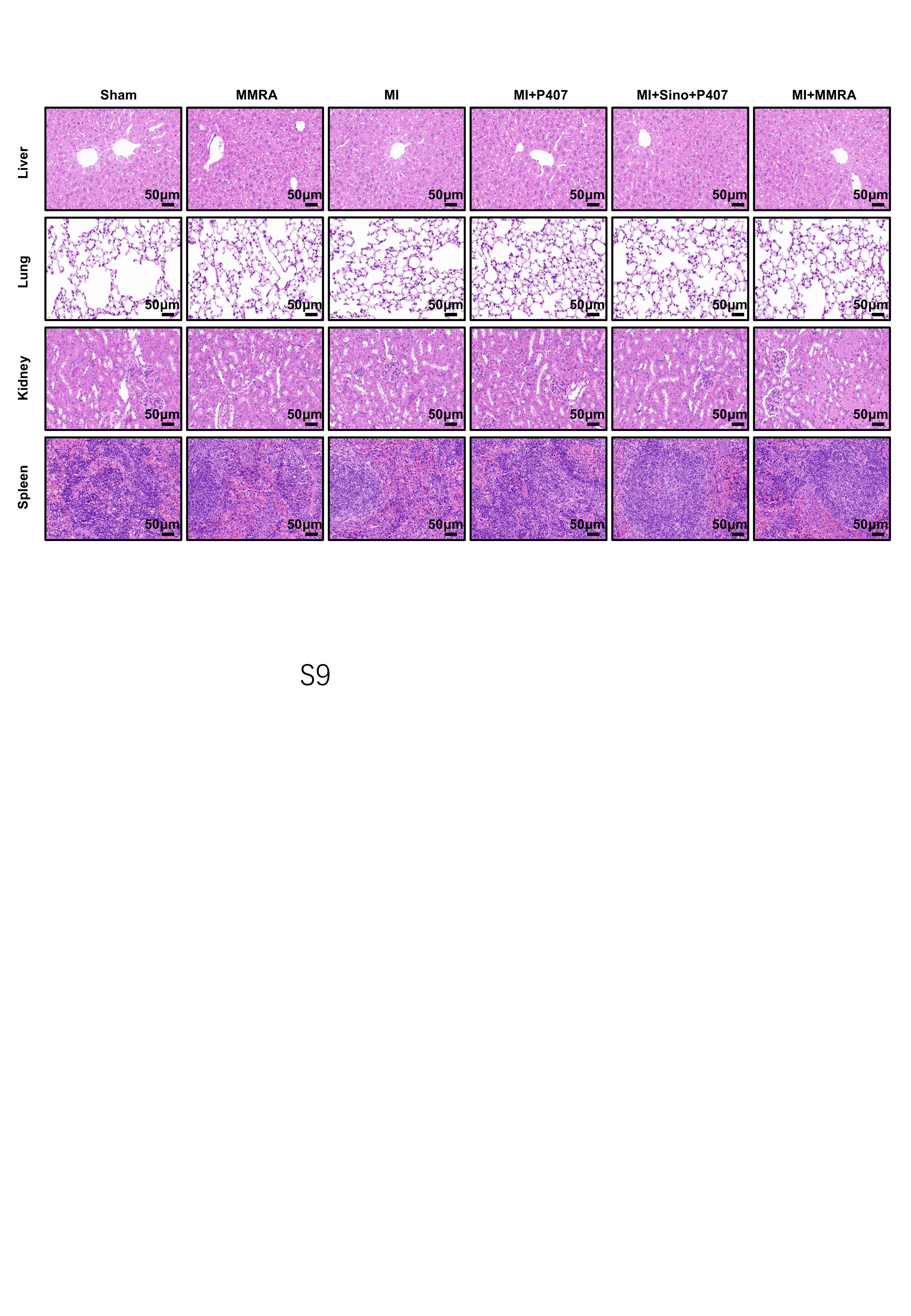


**Fig. S8.** Representative H&E staining of major organs from mice. Representative images of liver, lung, kidney and spleen sections from different experimental groups are shown.


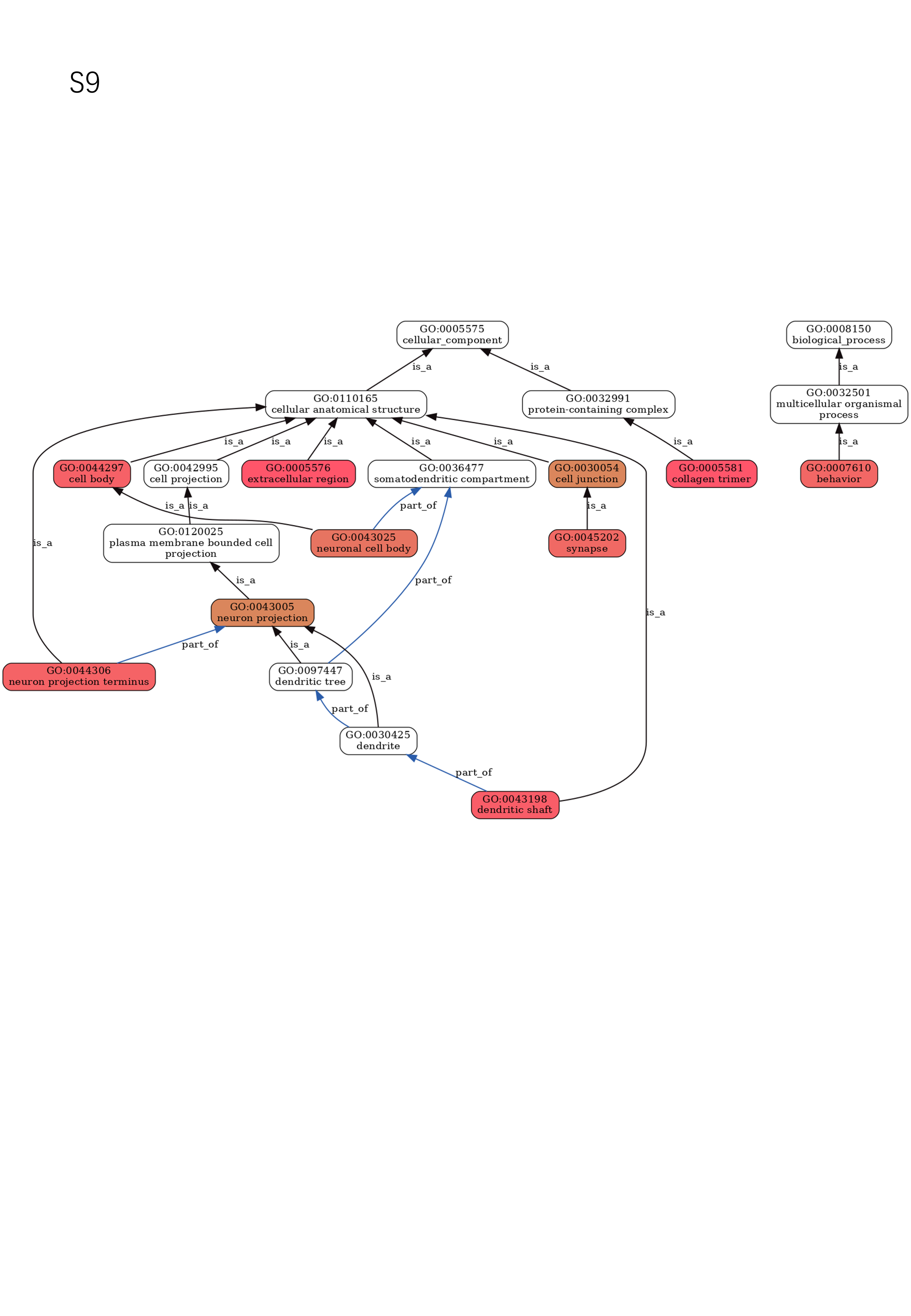


**Fig. S9.** GO directed acyclic graph (DAG) of significantly enriched GO terms (n=5).

**Fig. S10.** KEGG chord plot illustrating the relationship between DEGs and enriched pathways (n=5).
